# Does the Cosmopolitan Diatom *Gomphonema parvulum* (Kützing) Kützing Have a Biogeography?

**DOI:** 10.1371/journal.pone.0086885

**Published:** 2014-01-29

**Authors:** Nelida Abarca, Regine Jahn, Jonas Zimmermann, Neela Enke

**Affiliations:** 1 Botanischer Garten und Botanisches Museum Berlin-Dahlem, Freie Universität Berlin, Berlin, Germany; 2 AG Spezielle Botanik, Justus-Liebig-Universität, Giessen, Germany; Australian Museum, Australia

## Abstract

Diatom cultures of the *G. parvulum* species complex were established from seven different sites in the Faroe Islands, Sweden, Germany, Mexico and Korea, and were studied in detail. Eight morphodemes were identified which corresponded to the descriptions of the cosmopolitan taxon *G. parvulum* (Kützing) Kützing sensu lato: its nominate variety (var. *parvulum*), *G. parvulum* var. *exilissimum* Grunow and *G. parvulum* f. *saprophilum* Lange-Bertalot & Reichardt, *G. [parvulum* var.] *lagenula* Kützing plus four unidentifiable morphodemes. The concatenated analysis of the sequences of the markers 18SV4, *rbc*L, and ITS as well as morphological data resulted in a separation of four taxa based on their biogeography in Mexico, Korea, central Continental Europe and Northern Atlantic Europe. Mantel tests showed a significant correlation between molecular and geographical distances. The diagnoses of two taxa, *G. parvulum sensu stricto*, and *G. lagenula*, were emended, G. *saprophilum* elevated to species rank and epitypes designated. One species was newly described.

## Introduction

Diatoms are eukaryotic unicellular microalgae present in all types of water bodies and therefore often considered to be cosmopolitan and even ubiquitous. With their cell sizes ranging mainly between 10 µm and 200 µm, they seem to fit the hypothesis that microorganisms smaller than 1 mm have a cosmopolitan distribution in which the environment – here the ecology of the water – determines the occurrence of a taxon [1], [2], [3]. Recent studies on diverse protists [4], [5], [6], [7], [8], [9] and on different groups of diatoms revealed that, while some taxa are endemic for certain regions, islands or continents, others are more widely distributed [10], [11], [12], [13], [14]. For larger “flagship taxa” specific occurrences are more easily recognized than for small and inconspicuous species [15]. Although a general pattern of diatom biogeography remains to be discovered, some general ideas on the comparability of the floristic realms of diatoms and higher plants, and the cosmopolitan distribution of the most common species, to which our studied taxon *Gomphonema parvulum* belongs, have been discussed by Lange-Bertalot [16]. But foremost it seems that the quality and in-depth identification is key to a true and stable distribution record forming the basis for biogeography. The identification of diatoms relies mainly on micro-morphological characters which require in-depth knowledge of diatom taxonomy. If diatom diversity assessments are not done by experts of this taxon, the results are often misidentifications [17] and therefore misinterpretations of their distribution.

Molecular methods are playing an increasingly important role in the discovery and delimitation of diatom species and are revealing a hidden diversity, i.e. many taxa contain cryptic species, resulting in the description of many more species [18], [19], [20], [13]. Especially in cases where micro-morphological characters are insufficient for species delimitation, molecular data can provide additional characters and evidence for cryptic species. Several studies [21], [22], [14], [23] have shown that the molecular-phylogenetic analyses of diatom genera can support previous morphological observations. But there is also evidence that molecular sequence data sets yield significantly increased levels of taxonomic resolution compared to analyses of morphological data alone [24].

*Gomphonema parvulum* (Kützing) Kützing is a slender diatom of about 25 µm length and 6 µm width. It has long been recognized as a cosmopolitan, ubiquitous, and morphologically highly variable taxon for which many varieties have been described [25], [26], [27], [28], [29], [30], [31], [32], [33], [34], [35], [36], [37], [38], [39], [40], [41], [42] Taxonomic discrimination between morphologies is hard to disentangle because of similarities between species, supposed transitional forms, and/or high infra-specific variation. The current separation is based on micro-morphological characters of the valve, e.g., the pattern of the central area, density and branching of the striae, the form of the punctae, as well as the outline as revealed by light and scanning electron microscopy [43]. At the time of writing, Algaebase (www.algaebase.org) includes 28 taxon names with 23 varieties and 4 forms of which 12 are currently accepted taxonomically. *Gomphonema parvulum s.l.* has been reported worldwide as an abundant benthic species occurring over a wide range of freshwater environmental conditions, including a few saline lakes and brackish waters. In addition, cells from the *Gomphonema parvulum* group seem to respond sensitively to water-quality changes. Some (infra-specific) taxa in this species complex, therefore, were identified as indicators of environmental conditions in both ecological and paleoecological studies [23], [40], [44], [45], [46], [47], [48], [49]

The aim of this study is to determine whether taxa of the *Gomphonema parvulum* group from different ends of the Eurasian holarctic region can be differentiated by their morphological and molecular data, and if these in turn are different to taxa from the neotropics. Besides possible biogeographical distribution patterns, given the tangled taxonomy of this group mentioned above, it is necessary to clarify if morphological identification is supported by molecular data, and to assess potential cryptic species, as well as infraspecific morphological variation. These questions are tested with a number of morphometric features and three molecular markers on 21 cultures from 7 sites from the Faroe Islands, Sweden, Germany, Mexico and Korea. The recently-published paper and its available molecular and morphological data for several *Gomphonema parvulum* strains [50] will be compared to our data.

## Materials and Methods

### Field collection and cultures

**Figure 1:**
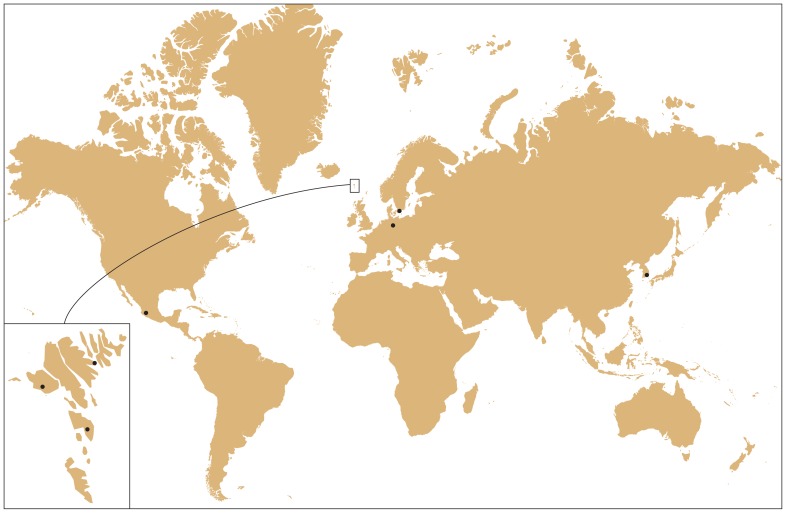
World map with sampling sites indicated by black dots.

Freshwater samples were collected from Northern Germany, Southern Sweden, Faroe Islands, Korea, and Mexico between 2004 and 2005 (Table 1, Fig. 1). The water samples were taken from public water bodies, no location was on protected or private land. No permits were required for the described study, which complied with all relevant regulations. The field studies did not involve endangered or protected species. 21 uni-algal cultures of *Gomphonema parvulum* taxa were established by micropipette isolation, for details see Romero & Jahn [51]. For morphological and genetic characterisation, cells were harvested in exponential phase and concentrated by centrifugation.

**Table 1:** Name, voucher number, origin, date, collector as well as Genbank Accession numbers of the studied strains.

Strain	Voucher at BGBM	Taxonname	Origin, date and collector	ITS	18 S	rbCl
D03_167	B40 0040900	*Gomphonema saprophilum* (Lange-Bertalot & Reichardt) Abarca et al. comb. nov.	Germany, Berlin, River Spree, Lat 52.49491° N, Lon 13.44729° 22.03.2004, ±20 m, O. Skibbe	-	HG529999	HG530036
D12_022	B40 0040901	*Gomphonema parvulum* (Kützing) Kützing var. *parvulum* [morphodeme *exilissimum*]	Denmark, Faroe Islands, Vágar, outlet of Leitisvatn, Lon 62.024485°, Lat −7.249842° ±2000 m, 08.08.2004, J. Bansemer	HG530018	HG530000	HG530037
D13_034	B40 0040902	*Gomphonema parvulum* (Kützing) Kützing var. *parvulum*	Denmark, Faroe Islands, Sandoy, at Skarvanes, Lake Stóravatn, Lon 61.816427°, Lat −6.73183° ±2000 m, 10.8.2004, J. Bansemer	-	-	HG530038
D16_004	B40 0040903	*Gomphonema parvulum* (Kützing) Kützing var. *parvulum*	Denmark, Faroe Islands, Viöoy, Waterfall below church, Lat 62.360335°, Lon −6.542833° ±20 m, 04.08.2004, J. Bansemer	HG530019	HG530001	HG530039
D16_005	B40 0040904	*Gomphonema parvulum* (Kützing) Kützing var. *parvulum*	Denmark, Faroe Islands, Viöoy, Waterfall below church, Lat 62.360335°, Lon −6.542833° ±20 m, 04.08.2004, J. Bansemer	HG530020	HG530002	HG530040
D16_008	B40 0040905	*Gomphonema parvulum* (Kützing) Kützing var. *parvulum*	Denmark, Faroe Islands, Viöoy, Waterfall below church, Lat 62.360335°, Lon −6.542833° ±20 m, 04.08.2004, J. Bansemer	HG530021	HG530004	HG530041
D16_009	B40 0040906	*Gomphonema parvulum* (Kützing) Kützing var. *parvulum*	Denmark, Faroe Islands, Viöoy, Waterfall below church, Lat 62.360335°, Lon −6.542833° ±20 m, 04.08.2004, J. Bansemer	HG530022	HG530003	HG530042
D16_011	B40 0040907	*Gomphonema parvulum* (Kützing) Kützing var. nov.?	Denmark, Faroe Islands, Viöoy, Waterfall below church, Lat 62.360335°, Lon −6.542833° ±20 m, 04.08.2004, J. Bansemer	HG530023	-	HG530043
D16_026	B40 0040908	*Gomphonema parvulum* (Kützing) Kützing var. nov.?	Denmark, Faroe Islands, Viöoy, Waterfall below church, Lat 62.360335°, Lon −6.542833° ±20 m, 04.08.2004, J. Bansemer	-	HG530005	HG530044
D16_027	B40 0040909	*Gomphonema parvulum* (Kützing) Kützing var. *parvulum*	Denmark, Faroe Islands, Viöoy, Waterfall below church, Lat 62.360335°, Lon −6.542833° ±20 m, 04.08.2004, J. Bansemer	HG530024	HG530006	HG530045
D16_028	B40 0040910	*Gomphonema parvulum* (Kützing) Kützing var.nov.?	Denmark, Faroe Islands, Viöoy, Waterfall below church, Lat 62.360335°, Lon −6.542833° ±20 m, 04.08.2004, J. Bansemer	HG530025	HG530007	HG530046
D16_030	B40 0040911	*Gomphonema parvulum* (Kützing) Kützing var. *parvulum*	Denmark, Faroe Islands, Viöoy, Waterfall below church, Lat 62.360335°, Lon −6.542833° ±20 m, 04.08.2004, J. Bansemer	HG530026	HG530008	HG530047
D16_042	B40 0040912	*Gomphonema parvulum* (Kützing) Kützing var. *parvulum*	Denmark, Faroe Islands, Viöoy, Waterfall below church, Lat 62.360335°, Lon −6.542833° ±20 m, 04.08.2004, J. Bansemer	HG530027	HG530009	HG530048
D16_044	B40 0040913	*Gomphonema parvulum* (Kützing) Kützing var. *parvulum*	Denmark, Faroe Islands, Viöoy, Waterfall below church, Lat 62.360335°, Lon −6.542833° ±20 m, 04.08.2004, J. Bansemer	HG530028	HG530010	HG530049
D16_045	B40 0040914 EPITYPE	*Gomphonema parvulum* (Kützing) Kützing var. *parvulum*	Denmark, Faroe Islands, Viöoy, Waterfall below church, Lat 62.360335°, Lon −6.542833° ±20 m, 04.08.2004, J. Bansemer	HG530029	HG530011	HG530050
D20_027	B40 0040915	*Gomphonema saprophilum* (Lange-Bertalot & Reichardt) Abarca et al. comb. nov.	Schweden, Bay Skälderviken Shore at Skepparkroken, freshwater rivulet, Lat. 56.281186, Long. 12.827908 ±100 m, 0 m a.s.l., 04.08.2004, R. Jahn	HG530030	HG530012	HG530051
D23_009	B40 0040916	*Gomphonema narodoense* R.Jahn et al. sp. nov.	Korea; ChollaNamdo, NaeNarodo Island, Spring, Lat 34.533042° Lon 127.463672 ±50 m, 114 m asl, 14.10.2004, R. Jahn	HG530031	HG530013	HG530052
D23_012	B40 0040917 HOLOTYPE	*Gomphonema narodoense* R.Jahn et al. sp. nov.	Korea; ChollaNamdo, NaeNarodo Island, Spring, Lat 34.533042° Lon 127.463672 ±50 m, 114 m asl, 14.10.2004, R. Jahn	HG530032	HG530014	HG530053
D33_006	B40 0040694	*Gomphonema lagenula* Kützing	Mexico, Ixtlán de los Hervores, Duero River, Lat. 20.147014, Long. −102.397240 ±20 m, Alt. 1534 m a.s.l, 06.12.2004, N. Abarca	HG530033	HG530015	HG530054
D33_024	B40 0040918 EPITYPE	*Gomphonema lagenula* Kützing	Mexico, Ixtlán de los Hervores, Duero River, Lat. 20.147014, Long. −102.397240 ±20 m, Alt. 1534 m a.s.l, 06.12.2004, N. Abarca	HG530034	HG530016	HG530055
D36_003	B40 0040919 EPITYPE	*Gomphonema saprophilum* (Lange-Bertalot & Reichardt) Abarca et al. comb. nov.	Germany, Berlin, Tiergarten, Canal Landwehrkanal, Lat 52.511°, Lon 13.339°, ±20 m, 11.06.2005, W.-H. Kusber	HG530035	HG530017	HG530056

### DNA isolation

The harvested cultures were transferred to 1.5 ml tubes. DNA was isolated using either Dynal® DynaBeads (Invitrogen Corporation; Carlsbad, CA, USA) or Qiagen® Dneasy Plant Mini Kit (Qiagen Inc.; Valencia, CA) following the respective product instructions. DNA concentrations were checked using gel electrophoresis (1.5% agarose gel) and Nanodrop® (PeqLab Biotechnology LLC; Erlangen, Germany). DNA samples were stored at −20°C until further use. Long-term storage of samples will take place at −80°C within the DNA bank network [52].

### PCR amplification

The samples were all amplified with the primer pair M13F-D512for 18S/M13F-D978rev 18S [53] for the V4 region of the 18S locus and the primer pair ITS1/ITS4 [54] for the ITS region. The *rbc*L locus was amplified in two overlapping parts using two different primer pairs; Diat-rbcL-F (5′-AAGTGACCGTKACGAATCTGG-3′) and Diat-rbcL-iR (5′-AAGAAWCGYTCTCTCCAACG-3′) as well as Diat-rbcL-iF (5′-AAGGHTTAAAAGGTGGTTTAGA-3′) and Diat-rbcL-R (5′-RTARAAACCTTTAATCA-3′). These primers were developed by Birgit Gemeinholzer at the BGBM. The polymerase chain reaction (PCR) for the V4 region was conducted following Zimmermann et al. [53]. For ITS the PCR mix (25 µl) consisted of 8.35 µl HPLC H_2_O, 2.5 µl 10× buffer S, 1.5 µl MgCl_2_, 5 µl pecGOLD dNTPs (each 1.25 mM), 2.5 µl Betaine (all products by PeqLab Biotechnology), 2 µl of each primer (20 pm/µl), 0.15 µl HotStarTaq® polymerase (Qiagen), and 1 µl DNA sample. Our ITS PCR regime followed Amato et al. [55] except for an increased annealing temperature of 48°C. For *rcb*L the PCR mix (25 µl) consisted of 12.9 µl HPLC H_2_O, 2.5 µl 10× buffer S, 1.5 µl MgCl_2_, 2.5 µl pecGOLD dNTPs (each 1.25 mM), 2.5 µl Betaine (all products by PeqLab Biotechnology), 1 µl of each primer (20 pm/µl), 0.15 µl HotStarTaq® polymerase (Qiagen), and 1 µl DNA sample. The PCR regime for *rbc*L initial denaturation 2 min at 94°C, 40 cycles of denaturation 1 min at 94°C, annealing 45 s at 52.8°C (rbcL-F/rbcL-iR) resp. 38.6°C (rcbL-iF/rbcL-R) and elongation 1 min 30 s at 72°C, followed by a final elongation of 10 min at 72°C.

PCR products were visualised in a 1.5% agarose gel and cleaned with MSB Spin PCRapace® (Invitek LLC; Berlin, Germany) following standard procedure. DNA content was measured using Nanodrop (PeqLab Biotechnology). The samples were normalised to a total DNA content >200 ng using the spectrophotometer Nanodrop® ND1000 (PeqLab Biotechnology).

### Sequencing

The Sanger sequencing was conducted by Starseq® (GENterprise LLC; Mainz, Germany). As sequencing primers M13 tails were used for the V4 region, following Ivanova et al. [56]. The sequencing for ITS was conducted with the same primers used for the amplification.

The sequences were edited in ChromasPro (Technelysium Pty. Ltd.; Tewantin, Australia) aligned using ClustalW [57], and manually improved in BioEdit [58]. For part of the sequences the alignment was done in PhyDe® [59].

### Acquisition of morphometric data

Diatom frustules were cleaned in the laboratory at 80°C in H_2_O_2_. The hydrogen peroxide was removed by repeated centrifugation and washing events using distilled water. Cleaned subsamples were dispersed on cover glasses, dried, and embedded in the diatom resin Naphrax® for further microscopic observation. Photomicrographs of the cleaned valves were taken, using a Zeiss Axioscope-microscope with Differential Interference Contrast (DIC) and an AXIOAM MRc camera. The morphology of cleaned frustules was examined and photographed using both LM and scanning electron microscopy (SEM Philips 515).

Identification of the cleaned valves was based on Krammer & Lange-Bertalot [60], Lange-Bertalot [41], Lange-Bertalot & Metzeltin [61]. Voucher slides (see Table 1) of all cultures are kept in the BGBM-Berlin and are documented in the AlgaTerra Information System.

**Figure 2:**
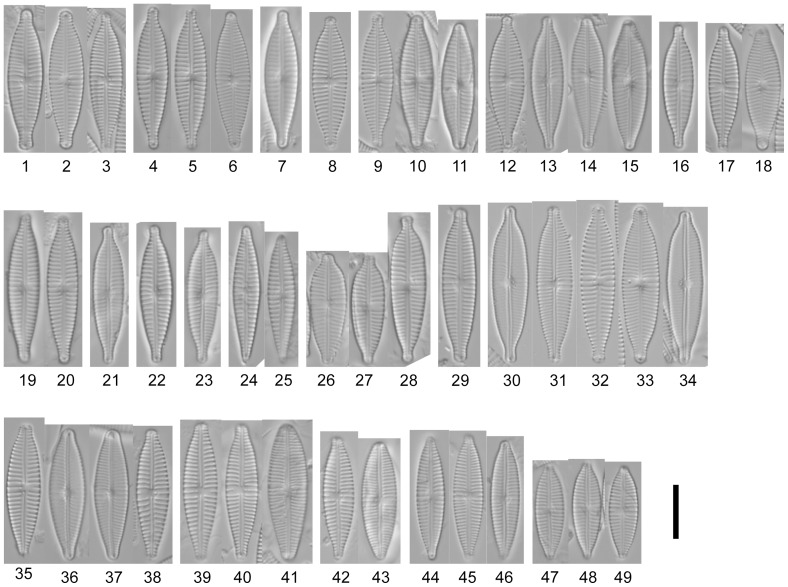
LM photos of strains. Figs. 2.1–22. *Gomphonema parvulum* (Kützing) Kützing var. *parvulum*. Figs. 2.1–3. Strain D16_042, Figs. 2.4–6. Strain D16_044, Fig. 2.7. Strain D16_005, Fig. 2.8. Strain D16_009, Figs. 2.9–11. Strain D16_008, Figs. 2.12–15. Strain D16_004, Figs. 2.16–18. EPITYPE Strain D16_045. Figs. 2.19–20. Strain D13_034, Fig. 2.21. Strain D16_030, Fig. 2.22. Strain D16_027. Figs. 2.23. Strain D16_026, *Gomphonema parvulum* var. nov.? Figs. 2.24–25. Strain D12_022, *Gomphonema parvulum* var. *parvulum* [morphodeme exilissimum]. Figs. 2.26–28. *Gomphonema lagenula* Kützing. Figs. 2.26–27. Strain D33_006. Fig. 2.28. EPITYPE Strain D33_024. Figs. 2.29–34. *Gomphonema parvulum* var. nov.? Figs. 2.29. Strain D16_028. Figs. 2.30–34. Strain D16_011. Figs. 2.35–43. *Gomphonema saprophilum* (Lange-Bertalot & Reichardt) Abarca et al. comb. nov. Figs. 2.35–38. EPITYPE Strain D36_003. Figs. 2.39–41. Strain D20_027, Figs. 2.42–43. Strain D03_167. Figs. 2.44–49. *Gomphonema narodoense* R. Jahn et al. sp. nov. Figs. 2.44–46. HOLOTYPE Strain D23_012. Figs. 2.46–49. Strain D23_009. Scale bars represent 10 µm.

**Figure 3:**
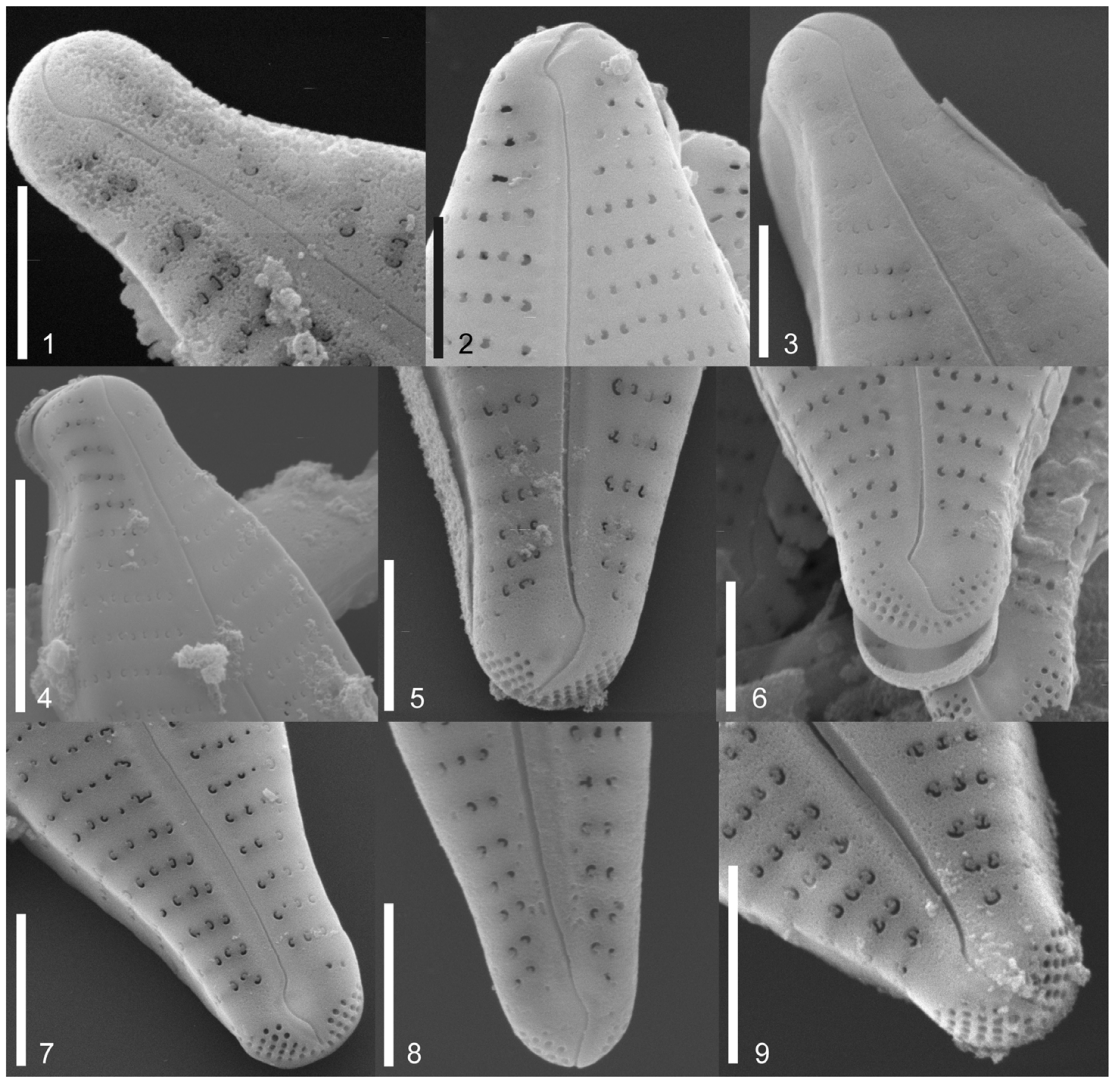
SEM morphology of studied strains; external valve view. Frustule outline. Apices: rostrate (Fig. 3.1), capitate (Fig. 3.4) and subrostrate to subcapitate (Fig. 3.2). External raphe fissure: head pole: hooked (Fig. 3.1), strongly hooked (Fig. 3.2), undulate (Fig. 3.3), slightly deflected (Fig. 3.4) and foot pole: hooked (Fig. 3.5), strongly hooked (Fig. 3.6), undulate (Fig. 3.7), slightly deflected (Fig. 3.8, 3.9). Fig. 3.1. Strain D16_026, *Gomphonema parvulum* var. nov.?. Fig. 3.2. Strain D16_008, *Gomphonema parvulum* var. *parvulum*. Fig. 3.3. Strain D13_034, *Gomphonema parvulum* var. *parvulum*. Fig. 3.4. EPITYPE Strain D33_024, *Gomphonema lagenula*. Fig. 3.5. Strain D20_027, *Gomphonema saprophilum*. Fig. 3.6. Strain D16_008, *Gomphonema parvulum* var. *parvulum*. Fig. 3.7. Strain D16_042, *Gomphonema parvulum* var. *parvulum*. Fig. 3.8. Strain D12_022, *Gomphonema parvulum* var. *parvulum* [morphodeme exilissimum]. Fig. 3.9 Strain D23_009, *Gomphonema narodoense* R. Jahn et al. sp. Nov. Scale bars usually represent 2 µm; the bar for Fig. 3.4 represents 5 µm.

**Figure 4:**
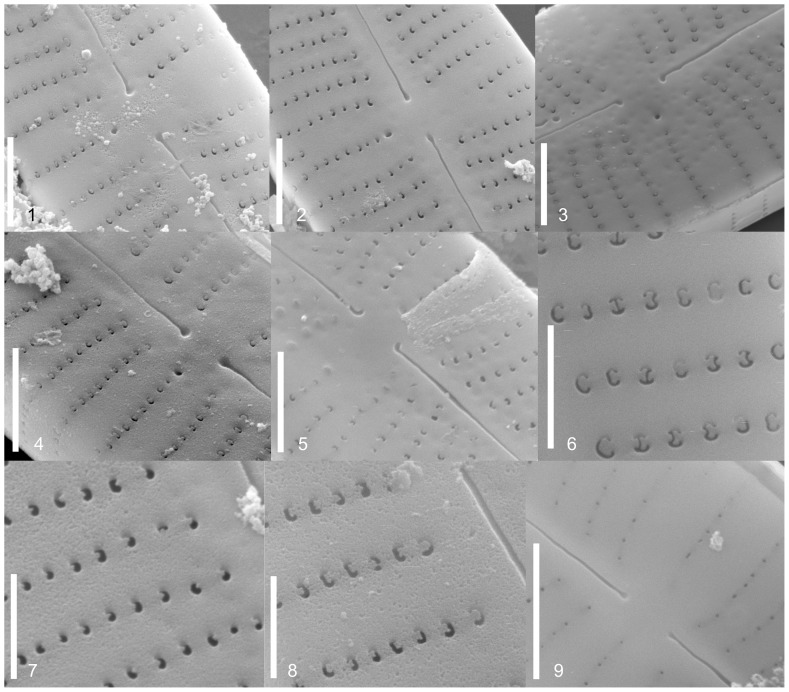
SEM morphology of studied strains, external valve view. Central raphe ending: droplike (Fig. 4.1), straight (Fig. 4.2), droplike deflected (Fig. 4.3), droplike spatulate (Fig. 4.4), hooked (Fig. 4.5). Areolae open: small rounded pores (Fig. 4.9), C shape (Fig. 4.3), E, 3 and anchor-like (Fig. 4.6), C, E, 3 (Fig. 4.8), kidney-shaped (Fig. 4.7). Alternating striae in the middle: short striae (Fig. 4.1), 2 short striae (Fig. 4.2), absent (Fig. 4.3). Fig. 4.1. Strain D16_030, *Gomphonema parvulum* var. *parvulum*. Fig. 4.2. Strain D16_042, *Gomphonema parvulum* var. *parvulum*. Fig. 4.3. Strain D16_011, *Gomphonema parvulum* var. nov.? Fig. 4.4. Strain D16_026, *Gomphonema parvulum* var. nov.? Fig. 4.5. Strain D16_008, *Gomphonema parvulum* var. *parvulum*. Fig. 4.6. HOLOTYPE Strain D23_012, *Gomphonema narodoense*. Fig. 4.7. Strain D16_044, *Gomphonema parvulum* var. *parvulum*. Fig. 4.8. Strain D20_027, *Gomphonema saprophilum*. Fig. 4.9. Strain D23_009, *Gomphonema narodoense*. Scale bars usually represent 2 µm; only in Figs. 4.6, 4.7 and 4.8 they represent 1 µm.

Valve length, width, density of striae and density and number of areolae of at least 10 valves per strain were measured using ImageJ 1.37v software (Table 2). The valves were screened for areolae openings, central raphe endings, alveoli and stigma openings. Table 3 gives an overview of the 11 frustule characters. The different characters are described with micrographs in Figs. 2, 3, 4; terminology follows Ross et al. [62] and Round et al. [43].

**Table 2:** Morphometrics of the studied strains.

Strain	Taxonname	Length (µm)^1^	Width (µm)	striae per 10 µm	Figs
D03_167	*Gomphonema saprophilum*	22–23	6–7	14–21	2.42–43
D12_022	*Gomphonema parvulum* var. *parvulum* [morphodeme *exilissimum*]	24–26.5	5–6	13–15	2.24–25, 3.8, 5.5
D13_034	*Gomphonema parvulum* var. *parvulum*	27.5–28	5–6	12–15	2.19–20, 3.3
D16_004	*Gomphonema parvulum* var. *parvulum*	24.5–25	6–6.7	15–18	2.12–15
D16_005	*Gomphonema parvulum* var. *parvulum*	24.5–26	6–7	14–15	2.7
D16_008	*Gomphonema parvulum* var. *parvulum*	23–25	6–7	14–18	2.9–11, 3.2, 3.6, 4.5
D16_009	*Gomphonema parvulum* var. *parvulum*	23–24.5	5.9–6.9	14–17	2.8
D16_011	*Gomphonema parvulum* var. nov?	28–29	6.5–7.5	14–19	2.30–34
D16_026	*Gomphonema parvulum* var. nov?	25–26	6–6.5	14–17	2.23, 3.1, 4.4, 5.4
D16_027	*Gomphonema parvulum* var. *parvulum*	24–25	5–6.5	15–17	2.22
D16_028	*Gomphonema parvulum* var. nov?	28–29	6–7	14–15	2.29, 5.7
D16_030	*Gomphonema parvulum* var. *parvulum*	25–26	5–6	15–16	2.21, 4.1
D16_042	*Gomphonema parvulum* var. *parvulum*	25.5–27	5–6	14–18	2.1–3, 3.7, 4.2
D16_044	*Gomphonema parvulum* var. *parvulum*	25–26.7	5–6.8	14–20	2.4–6, 4.7
D16_045	*Gomphonema parvulum* var. *parvulum*	22.8–24	5.7–7	13–18	2.16–18
D20_027	*Gomphonema saprophilum*	25.5–27	6–7.5	13–17	2.39–41, 3.5, 4.8, 5.3
D23_009	*Gomphonema narodoense*	17–18.5	5–6	16–17	2.47–49, 3.9, 4.9, 5.2
D23_012	*Gomphonema narodoense*	22–24	5–6	16–18	2.44–46, 4.6
D33_006	*Gomphonema lagenula*	20–21.2	6–7.5	14–18	2.26–27, 5.6
D33_024	*Gomphonema lagenula*	27–28	6–7	13–16	2.28, 3.4
D36_003	*Gomphonema saprophilum*	24.5–26	6.5–8	14–20	2.35–38, 5.1

1 Morphometric measurements were performed at least on 10 oxidized specimens per culture.

**Table 3:** Morphological characters of the studied strains.

		Internal View Central raphe endings				Alveoly		Stigma				Areola					External View Central raphe ending					Path of external raphe fissure – head pole				Path of external raphe fissure – foot pole				Alternating striae in the middle			Striae orient centre		Striae orient apex		Apix		
Taxon	Strain	hooked	deflected	strongly hooked	parrot head sha.	oval elongated	rectang. struts	short slit	slit	sperm-shape	round	small round	C shape	in E, 3, anchor	in C, E, 3	kidney-shaped	droplike	straight	droplike deflect.	droplike sphatul.	hooked	hooked	strongly hoked	undulate	slightly deflected	hooked	strongly hooked	undulate	slightly deflected	absent	short	2 short	radiate	transverse	radiate	transverse	rostrate	capitate	subrost-subcap
***Clade 2***	D03_167	-	-	-	X	X	-	-	-	X	-	-	X	-	-	-	-	-	X	-	-	-	-	-	-	-	-	-	-	-	X	-	-	X	-	X	X	-	X
	D20_027	X	-	-	-	X	-	X	-	-	-	-	X	-	X	-	-	X	-	-	-	X	-	-	-	X	-	-	-	-	X	X	X	X	X	X	X	-	X
	D36_003	-	X	-	-	X	-	-	X	-	-	-	-	-	-	X	-	-	-	X	-	-	-	-	-	-	-	-	-	-	X	-	X	X	-	X	-	-	X
***Clade 1***	D12_022	X	-	-	X	X	-	-	-	X	-	-	X	-	X	-	X	-	-	-	-	X	-	-	-	-	-	-	-	-	X	-	-	X	-	X	-	-	X
	D13_034	-	-	-	X	X	-	-	X	-	-	-	X	-	-	-	-	X	-	-	-	-	-	X	-	X	-	-	-	-	X	-	-	X	-	X	X	-	-
	D16_004	-	-	-	-	-	-	-	-	-	-	-	-	-	-	-	-	-	-	-	-	-	-	-	-	-	-	-	X	-	X	X	X	X	-	X	X	-	X
	D16_005	-	-	-	-	-	-	-	-	-	-	-	-	-	-	-	-	-	-	-	-	-	-	-	-	-	-	-	-	-	X	-	-	X	-	X	X	-	-
	D16_008	X	-	-	-	X	-	X	-	-	-	-	X	-	-	X	X	-	-	-	X	-	X	-	-	X	X	-	-	-	X	X	X	-	X	X	X	-	X
	D16_009	-	X	-	-	X	-	X	-	-	-	-	X	-	-	X	X	-	X	-	-	-	-	-	-	X	-	-	-	-	X	X	X	X	X	X	X	-	-
	D16_011	X	-	-	-	X	X	-	X	-	-	-	X	-	-	-	-	-	X	-	-	-	-	-	-	X	-	-	-	X	X	-	X	X	X	X	X	-	-
	D16_026	-	-	X	X	X	X	-	-	X	-	-	X	-	-	-	-	-	-	X	-	X	-	-	-	X	-	-	-	X	X	-	X	X	X	X	X	-	-
	D16_027	-	-	-	-	-	-	-	-	-	-	-	-	-	-	-	-	-	-	-	-	-	-	-	-	-	-	-	-	-	X	-	X	X	-	X	X	-	-
	D16_028	-	-	X	-	X	-	X	-	-	-	-	X	-	X	-	-	X	X	-	-	X	-	-	-	X	-	-	-	-	X	-	-	X	-	X	X	-	-
	D16_030	X	-	-	-	X	-	-	X	-	-	-	X	-	X	-	X	-	-	-	-	-	-	-	-	-	-	-	-	-	X	-	X	-	-	X	X	-	-
	D16_042	-	X	-	-	X	-	-	X	-	-	-	X	-	-	X	-	X	-	-	-	X	-	-	-	-	-	X	-	-	X	X	X	X	-	X	X	-	-
	D16_044	X	-	-	-	X	-	-	X	-	-	-	-	-	-	X	X	-	-	-	-	-	X	X	-	X	-	-	X	-	X	-	X	X	-	X	X	-	-
	D16_045	-	-	-	-	X	-	-	-	-	-	-	-	-	-	X	-	-	-	X	-	-	-	-	-	-	-	-	-	-	X	-	X	X	-	X	X	-	X
***Clade 4***	D23_009	X	-	-	-	X	-	-	-	-	X	X	X	X	-	-	X	X	-	-	-	-	-	X	-	X	-	-	-	-	X	-	-	X	-	X	-	-	X
	D23_012	X	-	-	-	X	-	-	-	-	X	-	X	X	-	-	-	-	X	-	-	-	-	-	-	-	-	-	-	-	X	-	-	X	-	X	-	-	X
***Clade 3***	D33_006	-	-	X	-	-	X	-	X	-	-	X	X	-	-	-	-	-	X	-	-	-	-	-	-	-	-	-	-	-	X	-	X	X	X	-	X	-	-
	D33_024	-	-	X	-	-	X	-	X	-	-	-	X	-	-	-	X	-	-	-	-	-	-	-	X	-	-	-	X	-	X	-	X	X	X	-	-	X	-

### Phylogenetic analyses

The morphological data were coded in a 0/1 matrix including the characters given in Table 3 plus valve length and breadth as well as the number of striae per 10 µm given in Appendix S1/A. Categories in 0.5 µm spans were established for valve length and breadth. The range for each strain was then coded as absence/presence (0/1). The number of striae found in each strain was also coded comparably, the categories being the numbers of striae per 10 µm in 0.5 steps. The binary 0/1 coding was chosen to avoid assumptions concerning the homology of characters. Five different data sets were used for the phylogenetic analysis: morphological matrix, ITS, 18SV4 and *rbc*L individual datasets (trees given in Appendix S1/B). After establishing that the individual tree topologies were not conflicting (considering only nodes with bootstrap support >75) a concatenated dataset including the morphological matrix and all three molecular markers was formed. Each dataset was analysed using three different approaches (Maximum Parsimony MP, Maximum Likelihood ML, Bayesian Likelihood BL) except for the morphological matrix which was analysed using two different approaches (MP, BL).

To test the stability of the recovered clades in a broader context, all sequences for *Gomphonema parvulum* available from Genbank were downloaded (Table 4) [50]. Again, the individual datasets for each marker were first analysed and then an analysis on a concatenated dataset was run that included those strains from Genbank for which all three markers were available (Table 4) as well as all the 21 strains from the present study.

**Table 4:** *Gomphonema parvulum* strains from Genbank with accession number and country of origin.

		Genbank Accession Number		
Strain	Origin	18SV4	rbcL	ITS
-	Hungary	AJ243062	-	-
-	Kenya	-	JQ003567	-
LCRS221	New Zealand	JQ610159	JQ610167	-
TCC426	Mayotte	-	JQ354655	JQ354617
TCC428	Mayotte	-	JQ354656	JQ354618
TCC429	Mayotte	KC736624	JQ354657	JQ354619
TCC430	Mayotte	-	JQ354658	JQ354620
TCC431	Mayotte	-	JQ354659	JQ354621
TCC432	Mayotte	-	JQ354660	JQ354622
TCC433	Mayotte	-	JQ354661	JQ354623
TCC434	Mayotte	-	JQ354662	JQ354624
TCC436	Mayotte	-	JQ354663	JQ354625
TCC438	Mayotte	-	JQ354664	JQ354626
TCC439	Mayotte	-	-	JQ354627
TCC440	Mayotte	-	JQ354666	JQ354628
TCC447	Mayotte	-	JQ354667	JQ354629
TCC462	Mayotte	KC736625	-	-
TCC463	Mayotte	-	JQ354668	JQ354630
TCC464	Mayotte	-	JQ354669	JQ354631
TCC465	Mayotte	-	JQ354670	JQ354632
TCC466	Mayotte	-	JQ354671	JQ354633
TCC467	Mayotte	-	JQ354672	JQ354634
TCC470	Mayotte	-	JQ354673	JQ354635
TCC471	Mayotte	-	JQ354674	JQ354636
TCC473	Mayotte	-	JQ354675	JQ354637
TCC478	Mayotte	-	JQ354676	JQ354638
TCC482	Mayotte	-	JQ354677	JQ354639
TCC485	Mayotte	-	JQ354678	JQ354640
TCC492	Mayotte	KC736626	JQ354679	JQ354641
TCC494	Mayotte	-	JQ354680	JQ354642
TCC500^2^	Mayotte	-	JQ354681	JQ354643
TCC592	France mainland	-	JQ354683	JQ354644
TCC593	France mainland	-	JQ354684	JQ354645
TCC595	France mainland	KC736627	JQ354685	JQ354646
TCC610	France mainland	-	JQ354686	JQ354647
TCC612	France mainland	KC736628	JQ354687	JQ354648
TCC653	Luxembourg	-	JQ354688	JQ354649
TCC664	Luxembourg	JN790284	JQ354689	JQ354650
TCC683	Italy	-	JQ354690	JQ354651
TCC725	Portugal	JN790283	JQ354691	JQ354652
TCC734	Portugal	JN790286	JQ354692	JQ354653
TCC736	Portugal	JN790285	JQ354693	JQ354654
UTEXFD241	-	HQ912595	HQ912459	-

2 also registered as *G.* cf. *lagenula* in Genbank.

Prior to the phylogenetic analysis, the datasets were tested for their phylogenetic information content using SAMS [63] with standard options. First, the MP analysis was run on PAUP 4.0b10 [64] with equal weights, 10,000 closest sequence additions and tree bisection-reconnection (TBR) branch swapping, permitting 1000 trees to be held at each step. A strict consensus tree was computed. The trees were evaluated by a bootstrap analysis [65] with 10,000 replicates (using the same search strategy as the MP analysis) and MulTrees option in effect (but limiting the maximum tree number to 10,000). For the ML and BL analysis of the molecular datasets, the optimal model of sequence evolution that best fitted the sequence data was calculated under the hierarchical likelihood ratio test (hLRT) and selected according to the Akaike information criterion (AIC) using Modeltest 3.7 [66]. This resulted in the model GTR+G+I [67]. Second, an ML analysis was conducted using RAxML 7.0.4 [68], [69], ML search option (GTR+G+I) and 10,000 bootstrap replicates (model GTRCAT as implemented in RAxML for the rapid bootstrap algorithm). The RAxML run was not conducted on the morphological matrix. A third analysis was run on MrBayes 3.1.2 [70] using gamma distribution rate variation among sites and 10,000,000 generations of the MCMC chains in two independent runs of four chains apiece; otherwise the default parameters were used. Convergence of MCMC chains was assumed when the standard deviation of split frequencies was lower than 0.01. Every hundredth tree was sampled. The first 25,000 trees were discarded as burn-in; the rest was used to calculate a 50% majority rule consensus tree. The strict consensus tree of the MP analysis was compared to the 50% majority rule tree of the BL analysis, and the best ML tree found by RAxML. Trees were drawn using FigTree v1.2.2 [71] and Adobe Illustrator (Adobe Systems, San Jose, CA).

### Nomenclature

The electronic version of this article in Portable Document Format (PDF) in a work with an ISSN or ISBN will represent a published work according to the International Code of Nomenclature for algae, fungi, and plants, and hence the new names contained in the electronic publication of a PLOS ONE article are effectively published under that Code from the electronic edition alone, so there is no longer any need to provide printed copies. The online version of this work is archived and available from the following digital repositories: PubMed Central, LOCKSS. http://edocs.fu-berlin.de/docs/content/below/index.xml.

### Genetic distances

Uncorrected p-distances were calculated for each marker individually as well as a concatenated data set using PAUP 4.0b10 [64] Those parts of the alignment including missing data were excluded from the analysis as PAUP cannot discriminate between gaps and missing data when calculating uncorrected p-distances. Results are given in Appendix S1/C.

### Mantel tests

A matrix of direct line geographic distances (km) and genetic distances (uncorrected p distances; calculated using PAUP 4.0b10 [64]) between populations was created (data available from the corresponding author upon request). Mantel tests [72] were used to examine associations between geographic and genetic distances using R 2.7.1 [73] with 9999 permutations.

## Results

**Morphological characters (** **Figs. 2** **,** **3** **,** **4** **,** **5** **)**

**Figure 5:**
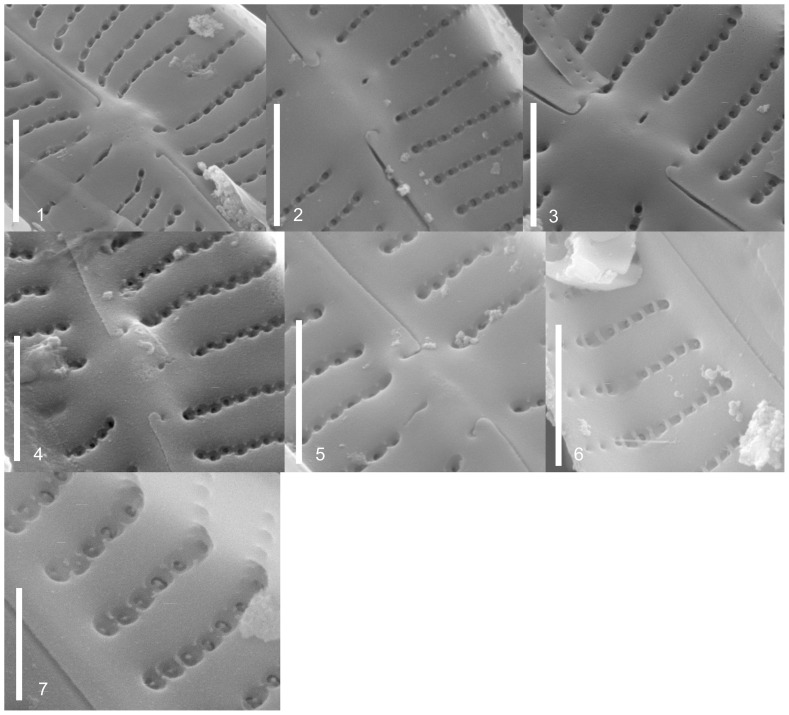
SEM morphology of studied strains, internal valve view. Central raphe endings: hooked (Fig. 5.2), deflected (Fig. 5.1), strongly hooked (Fig. 5.4) and parrot head-shape (Fig. 5.5). Alveoli: rectangular separated by silica struts (Fig. 5.6) and oval elongated separated by opened silica struts (Fig. 5.7). Stigma opening in internal view: short slit (Fig. 5.3), slit (Fig. 5.1), sperm-shape (Fig. 5.4) and round (Fig. 5.2). Scale bars represent 2 µm; only in Figs. 5.3 and 5.7 it represents 1 µm. Fig. 5.1. HOLOTYPE Strain D36_003, *Gomphonema saprophilum*. Fig. 5.2. Strain D23_009, *Gomphonema narodoense*. Fig. 5.3. Strain D20_027, *Gomphonema saprophilum*. Fig. 5.4. Strain D16_026, *Gomphonema parvulum* var. nov.? Fig. 5.5. Strain D12_022, *Gomphonema parvulum* var. *parvulum* [morphodeme exilissimum]. Fig. 5.6. Strain D33_006, *Gomphonema lagenula*. Fig. 5.7. D16_028, *Gomphonema parvulum* var.nov.?

The morphometric data (Table 2) and morphological features recorded for each strain (Table 3, Figs. 2, 3, 4, 5) showed the typical characteristics of *Gomphonema parvulum*: lanceolate or linear-lanceolate heteropolar valves with more or less subrostrate or rostrate heads. The central area is asymmetrical with a single long stria on one side terminating in a distinct stigma, and a single short stria on the other side. Striae are almost perpendicular to the raphe. Such characteristics, associated with the length (17–29 µm), breadth (5–8 µm), length/breadth ratio (3–5) and 12–21striae in 10 µm, allowed us to group all studied specimens into *Gomphonema parvulum* s.l.

In LM observations (Fig. 2), four of the eight different morphodemes that could be differentiated on the basis of their outline corresponded to named varieties and/or forms forms. These were: *parvulum sensu stricto* (D13_034, D16_004, D16_005, D16_008, D16_009, D16_027, D16_030, D16_042, D16_044, D16_045), *exilissimum* (D12_022), *lagenula* (D33_024, D33_006), *saprophilum* (D03_167, D20_027, D36_003). The remaining four morphodemes, as yet unnamed, did not correspond to named taxa. These morphodemes were (D16_028, D16_011, D16_026, D23_009 and D23_012).

The four unnamed morphodemes had a slightly different valve outline, making it difficult to assign them to any known group; the first two cannot be delimited convincingly because there seem to be transitions (*Übergangsformen*) between the outlines. Some of the proposed differentiating micro-morphological features are present within the same strain such as raphe fissures (Fig. 3), central raphe endings (Figs. 4 and 5), areolae (Fig. 4), alveoli (Fig. 5), stigma openings (Fig. 4), alternating striae in the center (Fig. 4),. For example, the areolae of the morphodeme *exilissimum* (Figs. 3.8 and 5.5) are supposed to be on average significantly larger than *G. parvulum* and have the shape of a horseshoe or ‘C’ as shown in Fig. 4.3 but the strain D12_22 shows large and small areolae with shapes including not only ‘C’ forms, but also ‘3’ and ‘E’. A further example is the morphodeme *saprophilum* which on average should have smaller areolae mostly comma to kidney-shaped but the strains D03_167, D20_027, D36_003 have a variety of forms and sizes such as ‘C’, ‘3’, ‘E’ and kidney-shapes (Fig. 4.8).

**Figure 6:**
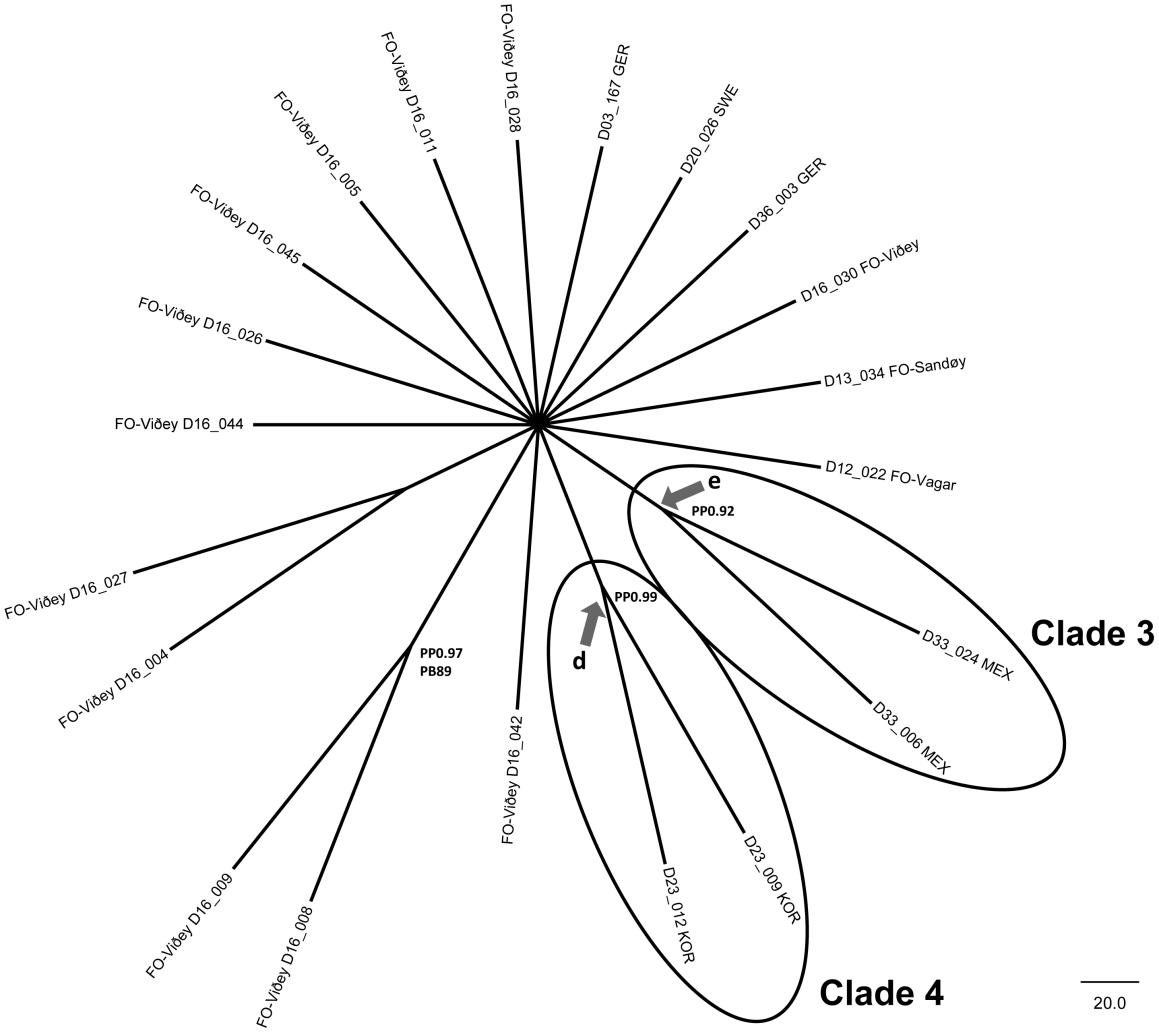
Strict consensus tree of the ML analysis of the morphological matrix with the results of the SAMS analysis (arrows with split names), bootstrap values (>75) of MP (PB) and ML (LB) as well as Bayesian posterior probabilities (PP) (>0.75).

**Cladistic analysis of morphological data** (Fig. 6)

Fig. 6 shows the strict consensus tree of the ML analysis of the morphological matrix (Table 2 and Table 3) with the results of the SAMS analysis, bootstrap values (>75) of MP (PB) and Bayesian posterior probabilities (>0.75, PP). Only 2 clades are well resolved; these were the two Mexican strains (Clade 3, Fig. 6, (PP 0.92) and the two Korean strains (Clade 4, Fig. 6, PP 0.99) which separated well from the rest of the strains. These two clades were the only ones that, following the SAMS analysis, were supported unambiguously by the data (shown by arrows in Fig. 6). In addition the sample pair D16_008 and D16_009 was also supported by BL (PP 0.97) as well as MP analysis (PB 89).

Since the morphometric data and the few differentiating morphological features overlap even within one strain, molecular data were used in order to identify and substantiate further differences between the strains.

**Figure 7:**
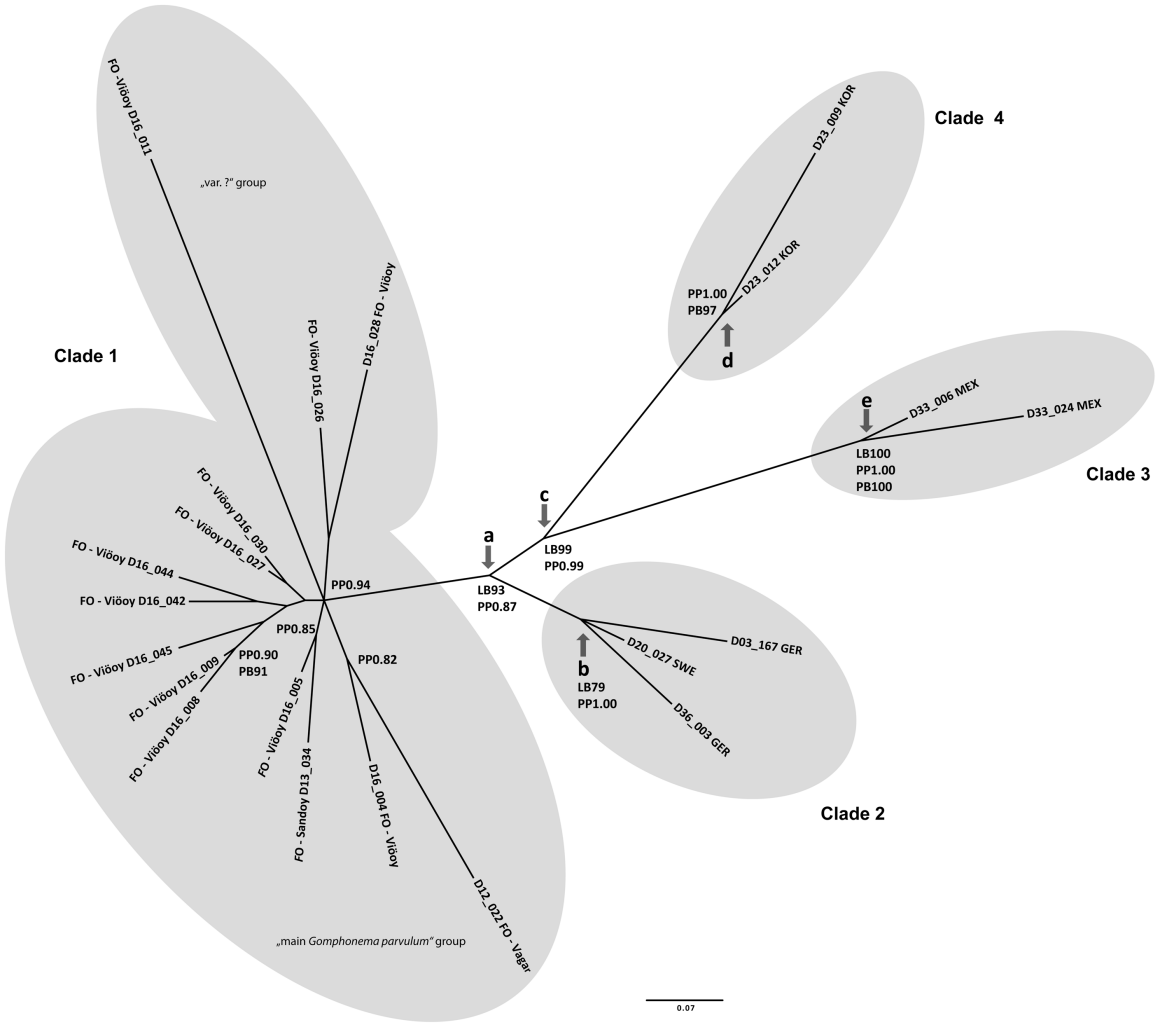
Strict consensus tree of the ML analysis of the combined dataset (molecular markers 18SV4, internal transcribed spacer ITS, *rbc*L and morphological matrix) with the results of the SAMS analysis (arrows with split names), bootstrap values (>75) of MP (PB) and ML (LB) as well as Bayesian posterior probabilities (PP) (>0.75).

**Figure 8:**
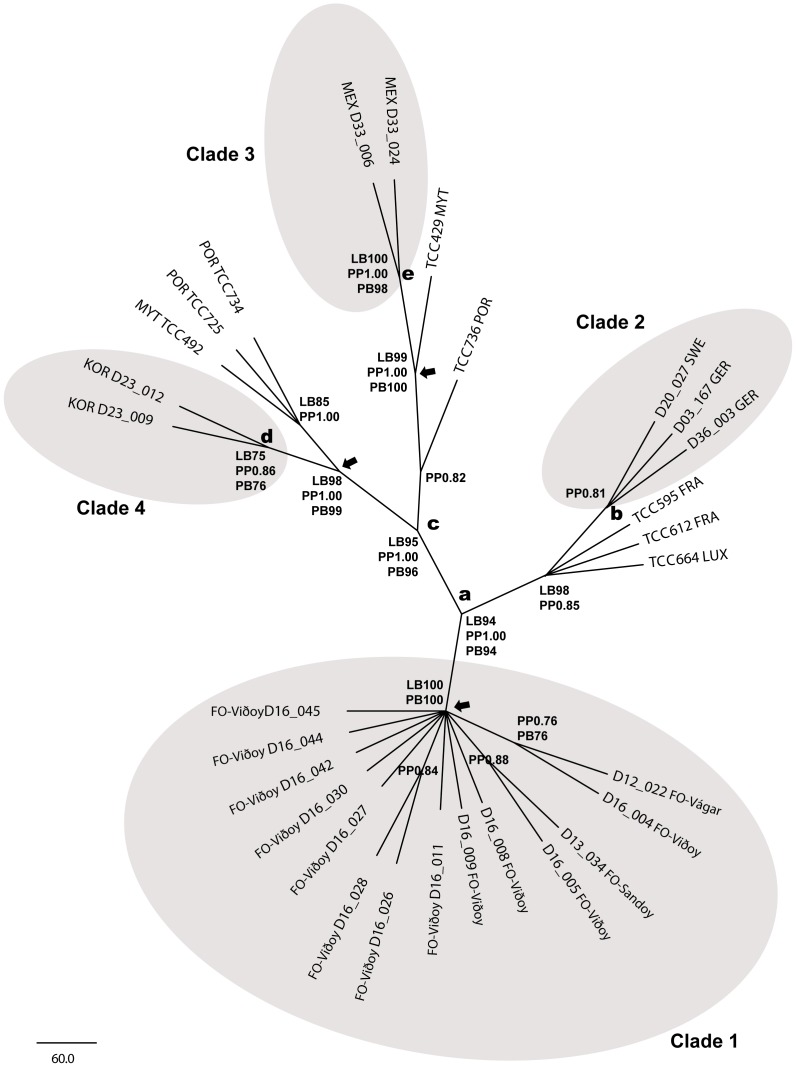
Strict consensus tree of the ML analysis of the combined dataset (molecular markers 18SV4, internal transcribed spacer ITS, and *rbc*L) including Genbank sequences with the results of the SAMS analysis (arrows), bootstrap values (>75) of MP (PB) and ML (LB) as well as Bayesian posterior probabilities (PP) (>0.75). Split names indicate the splits from Fig. 7.

**Phylogenetic analysis of molecular and combined data** (Fig. 7, Fig. 8)

The results of the phylogenetic analyses are summarised in Figs. 7 and 8. First the individual markers were analysed (trees are given in Appendix S1/B). In both approaches (excluding and including additional Genbank sequences) ITS and *rbc*L recovered non contradicting tree topologies (Appendix S1/B2, S1/B3, S1/B7, S1/B8). The analysis of 18SV4 recovered contradicting topologies (Appendix S1/B1, S1/B4, S1/B5, S1/B6); the main difference to the ITS and *rbc*L datasets being the position of strains D16_026 and D16_028 that cluster with D33_006 and D33_024 (e.g. Appendix S1/B1). However, the contradicting nodes are supported only by values below the threshold (>75 bootstrap support or >0.95 posterior probability), so 18SV4 could be included in the concatenated dataset.

Fig. 7 shows the strict consensus tree of the ML analysis of the combined dataset including only our own strains from the present study with the results of the SAMS analysis, bootstrap values (>75) of MP (PB) and ML (LB) as well as Bayesian posterior probabilities (>0.75, PP). As there are no outgroup taxa included in our analysis we chose an unrooted tree design. The SAMS analysis showed that the combined dataset comprising morphological characters and morphometric data (Tables 2 and 3) and the molecular markers (18SV4, *rbc*L and ITS) is the phylogenetically most informative dataset. The splits supported by the combined dataset are the same as in the individual subsets: however not all subsets support all splits. The splits that are unambiguously supported by the combined dataset following SAMS are indicated by arrows in Fig. 7. The supported splits are (a) the split between the strains from the Faroe Islands and the central Continental European, Mexican and Korean clones, (b) the split between the central Continental European clade and the rest of the clones, (c) the split between the Korean and Mexican clones and the rest, (d) the split between Korean clones and the rest and (e) the split between Mexican clones and the rest (Fig. 7).

The combined dataset shows the best resolved tree in comparison with the individual datasets. However, no contradictions are found in the other trees. In the following, all bootstrap values and posterior probabilities are given for the concatenated dataset if not stated otherwise. ML bootstraps (LB) are strong (>75) for the splits (a), (b), (c) and (e) (Fig. 7). BL posterior probabilities (PP) are high (>0.95) for splits (b), (c), (d) and (e); PP for split (a) is somewhat lower at 0.87 (Fig. 7). The splits (d) and (e) are also well (PB >75) supported by MP bootstraps (PB) (Fig. 7). The BL analysis also recovered three more sample pairs: D16_005 and D13_034 (PP 0.85), D16_008 and D16_009 (PP 0.90, also supported by MP analysis: PB 91) as well as D16_004 and D12_022 (PP 0.82) (Fig. 7). The long branch of D16_011 is due to missing data in the *rbc*L gene (Fig. 7).

Fig. 8 shows the outcome of the analysis of the concatenated dataset including additional Genbank sequences (Table 4). Clades 1–4 are recovered. Clade 1 is supported by ML (LB100) and MP (PB100, Fig. 8). Clade 2 is supported by BL (PP0.81), MP and ML recover a corresponding clade that includes D03_164, D20_027 and D03_036 as well as three further strains from France (TCC545, TCC612) and Luxembourg (TCC664, Fig. 8). The Mexican Clade 3 was supported by BL, ML and MP (PP1.00, LB100, PB98, Fig. 8) and was the sister group of a clade of strains from Mayotte (Appendix S1/B7, S1/B8; represented in Fig. 8 by strain TCC429). A strain from Portugal (TCC736) is sister to the strains from Mexico and Mayotte, however this is only weakly supported by BL (PP0.82). The Korean clade is supported by BL, ML and MP; however, not very strongly (PP0.86, LB76, PB75). The ITS and *rbc*L analysis show it is sister to another clade from Mayotte (Appendix S1/B7, S1/B8; represented in Fig. 8 by strain TCC492). In Fig. 8 there are also two strains from Portugal (TCC725, TCC734) clustering in this clade.

**Clade 1** is composed of strains from the Faroe Islands (Fig. 7). The strains showed a wide phenotypic plasticity, with specimens varying from isopolar to clearly heteropolar in shape, and with subrostrate, rostrate or subcapitate apical endings (Figs. 2.1–22). All of the strains present a mixture of valve characters such as dimensions, striae density, raphe characters, areolae, alveolae, stigma openings and even alternating striae in the center. This clade also includes the strain D12_022 which corresponds to the species description of *Gomphonema exilissimum* (Grunow) Lange-Bertalot & Reichardt 1996 in Lange-Bertalot & Metzeltin [61] and Jüttner et al. [23]; specimens have long, slender valves, are weakly clavate to linear-lanceolate with shortly protracted, rounded head poles and sharply narrowed foot poles (length 24–26.5 µm, width 5–6 µm, 13–15 striae in 10 µm) (Figs. 2.24, 2.25). There is no difference within a group of 7 strains across all three molecular markers (D16_030, D16_027, D16_044, D16_042, D16_045, D16_009, D16_008, Appendix S1/C4) and within a group of two strains (D16_005, D13_034, Appendix S1/C3, only available for *rbc*L, other markers too much data missing) and only very little (<0.2%, Appendix S1/C4) between all the strains of these two groups. The strains D16_004 and D12_022 show a little more difference to the above mentioned strains but stay below one percent (Appendix S1/C4). The three further strains of the Faroe Clade are difficult to compare since their data are not complete: The morphologically unidentified strain D16_028 (Fig. 2.29) shows a distance of about 1.3% in its 18SV4 (Appendix S1/C1) towards all the other strains from the Faroe Islands but it clusters closely with the strain D16_026 (Fig. 2.23); morphologically unidentified strain D16_011 (Figs. 2.30–34) shows clear differences in its ITS data of up to 4.6% to all the other strains of this clade (Appendix S1/C2); data for *rbc*L are incomplete and data for 18SV4 are missing.

As can be seen in Fig. 8, none of the additional Genbank sequences clustered in or close to our Clade 1.

**Clade 2** is composed of the two strains from Northern Germany (Berlin; D03_167, D36_003) and southern Sweden (Skepparkroken; D20_027) (Fig. 7); there are no differences in the 18SV4 data (Appendix S1/C1) and only 0.2% in the *rbc*L (Appendix S1/C3) and about 0.5% in the ITS (Appendix S1/C2). Morphologically, these three strains fit the concept of *Gomphonema parvulum* var. *parvulum* f. *saprophilum* [41].

In the analysis including additional Genbank sequences (Fig. 8) three more strains clustered in this clade originating from France (TCC545, TCC612) and Luxembourg (TCC664); in the corresponding paper by Kermarrec et al. [50], they belong to clade C which includes only strains from Europe (in that paper [50] the names clade C and clade D have been interchanged in the concatenated tree, we refer to the clade-naming in the single marker trees). Including these, the genetic distance over all three markers within this clade ranges between 0.2% and 0.3% (Appendix S1/C5). The highest variation is found in ITS where D36_003 differs by about 1% from TCC545, TCC612 and TCC664 (Appendix S1/C6).

**Clade 3** is composed of the two Mexican strains (Fig. 6, Fig. 7); the morphological data – apical ends subcapitate to capitate – fit the concept of *Gomphonema lagenula* (Figs. 2.26–28). In addition, the molecular data show very little variation: 0.3% for 18SV4 (Appendix S1/C1), none for *rbc*L (Appendix S1/C3).

Including the additional Genbank sequences (Fig. 8), there is a clade of strains from the tropical island Mayotte (Appendix S1/B7, S1/B8) that is represented in Fig. 8 by TCC429; in the corresponding paper [50], they have been identified as belonging to clade A which also includes only Mayotte strains. The genetic distance between the Mexican strains and TCC429 is 0.5% over all three markers (Appendix S1/C5), for *rbc*L there is no genetic difference between the Mexican strains and TCC429 (Mayotte), Another strain from Mayotte (TCC500), being identical with TCC429 in ITS and *rbc*L (Appendix S1/B7, S1/B8), has been identified as *G.* cf. *lagenula* by Kermarrec et al. [50].

The strain TCC736 from Portugal is neighbouring the Mexican and Mayotte strains (Fig. 8); the uncorrected p-distance between the Mexican clade and TCC736 is 1.3% over all three markers (Appendix S1/C5); for *rbc*L there is a genetic difference of 0.6% between these three and TCC736 (Portugal, Appendix S1/C7). In the corresponding paper [50], this strain is a representative of clade D (single marker trees in [50]) which is found in Europe only.

**Clade 4** is composed of the two Korean strains (Fig. 6, Fig. 7); the morphological data do not fit any of the described taxa; their outline is only slightly different from the other demes (Figs. 2.44–47) but is distinct enough to form a well supported clade even in the purely morphological analysis (Fig. 6). The molecular data show only little variation: none for 18SV4 and *rbc*L (Appendix S1/C1, S1/C3) and only 1.2% for ITS (Appendix S1/C2).

Including the additional Genbank sequences (Fig. 8), our Clade 4 forms the sister group to clade B and B′ from the corresponding paper [50], including strains from Mayotte (TCC492) and Portugal (TCC725, TCC734). The genetic differences over all three markers between the Korean clade and these strains range between 0.2% and 0.3% (Appendix S1/C5). However, if the markers are considered individually, there are some interesting patterns: there is no variation in 18S between all five strains (Appendix S1/C7). ITS shows no difference between TCC725, TCC734 (both Portugal) and TCC492 from Mayotte, with other differences ranging from 0.9% to 1.7%, the largest being from D23_012 to either TCC725 or TCC792 (Appendix S1/C7). Both Korean strains have an insert of 6 bp in ITS that is missing in the other three strains. For *rbc*L only TCC492 from Mayotte shows any difference (0.7%) from the other strains (Appendix S1/C7).

### Nomenclatural and taxonomical consequences

Because the molecular and morphological data of Kermarrec et al. [50] do not overlap with our study, the nomenclatural findings and taxonomical consequences of this section can only be based on our strains; but similarities will be pointed out in the discussion. The morphodeme *parvulum* (D16_004, D16_005, D16_008, D16_009, D16_027, D16_042, D16_044, D16_045) is restricted in our study to the Faroe Islands as can be seen in Fig. 6 and Fig. 7 as well as Fig. 8. The morphodeme *exilissimum* is found in only one culture on the Island Vagar (D12_022) on the Faroe Islands (Figs. 2.24–2.25, Fig. 7); it clusters within a subclade of the Faroe Islands clade together with a typical *parvulum* morphodeme (D16_004, Fig. 7), but this clade is not statistically supported. Three of the unidentified morphodemes (D16_028, D16_026, and D16_011) originate from three strains from the island Viöoy; they appear together in the same Faroe Islands subclade which is not supported (Fig. 7); in addition, some of their data are not complete (Table 1). These might represent varieties but we cannot solve their taxonomy. The fourth unidentified morphodeme is represented only in the Korean strains (D23_009, D23_012; Fig. 2.44–2.47, Fig. 7) which cluster together even morphologically (Fig. 6) and should be separated taxonomically with a name. The morphodeme *saprophilum* (D03_167, D20_027, D36_003) is found only in the northern central Continental European clade (Fig. 2.35–2.43, Fig. 7). The morphodeme *lagenula* (D33_024, D33_006) is observed only within the Mexican clade (Fig. 2.26–2.28, Fig. 6, Fig. 7). The above data show that the four unambiguous clades (Fig. 7) should be separated and named.

***Gomphonema parvulum*** **(Kützing) Kützing var.** ***parvulum*** (Figs. 2.1–22; 2.24–25; 3.2–3; 3.6–8; 4.1–2; 4.5; 4.7; 5.5)

Kützing, Species Algarum, p.65. 1849 [26].

Basionym: *Sphenella parvula* Kützing, Die kieselschaligen Bacillarien oder Diatomeen, p.83; taf. 30, fig. 63. 1844 [25].

Heterotypic synonyms: *G. parvulum* var. *exilissimum* Grunow**≡** *G. exilissimum* (Grunow) Lange-Bertalot & Reichardt 1996 [61], Figs. 22–27.

Lectotype designated here from the syntypes designated by Krammer & Lange-Bertalot [40] 1986: 358ff; fig. 154:2.

Type locality: Falaise, France.

Epitype (designated here): B 40 0040914 (strain D16_045); see Fig. 2.16.

Type locality of the epitype (designated here): Faroe Islands, Viöoy, Waterfall below church, Lat 62.360335°, Lon −6.542833° ±20 m, 04.08.2004, leg. J. Bansemer.

Emended species description:

This species is highly variable in its valve shape: lanceolate, linear-lanceolate or oval heteropolar valves with more or less subrostrate or rostrate head. The central area is asymmetrical with a single long stria on one side terminating in a distinct stigma, and a single short stria (sometimes two) on the other side. Striae slightly radiate or parallel, not distinctly punctuate. Length 22–29 µm, breadth 5–7.5 µm, striae 12–20 in 10 µm.

***Gomphonema saprophilum*** **(Lange-Bertalot & Reichardt) Abarca, R. Jahn, J. Zimmermann & Enke comb. nov.** (Figs. 2.42–43; 1.39–41; 2.35–38; 3.5; 4.8; 5.1; 5.3)

Basionym: *G. parvulum* var. *parvulum* f. *saprophilum* Lange-Bertalot & Reichardt 1993, Bibliotheca Diamologica 27, p. 69 [41]; figs. in Krammer & Lange-Bertalot [40] 1991, figs. 76: 8–13; not figs. 77: 5–9.

Heterotypic synonym?: *G. parvulum* var. *parvulum* f. *neosaprophilum*. Kobayasi et al. [42]

Lectotype (designated here): fig.76: 11 in Krammer & Lange-Bertalot 1991 [40]

Type locality: Kommunale Kläranlage der Stadt Frankfurt a.M.; “voll” gereinigtes Abwasser mit einer Restbelastung von BSB_5_-Werten um 20 mg O_2_/L (15.1.1975).

Epitype (designated here): B 40 0040919 (strain D36_003), see Fig. 2.35

Epitype locality: Germany, Berlin, Tiergarten, Landwehrkanal, Lat 52.511°, Lon 13.339°, ±20 m, 11.06.2005, leg. W.-H. Kusber.

Emended Diagnosis: This taxon is separable from *G. parvulum* based mostly on the valve-shape; the taxon *G. saprophilum* has a rather rhomboidal shape with slightly rostrate ends, the head pole and the valve are on average also wider than those of *G. parvulum* (Figs. 2.35–2.43). Valves are clavate-lanceolate, 22–27 µm long, and 6–8 µm wide; head poles shortly rostrate and foot poles obtuse or subcapitate. The axial area is linear and the central area is narrow. The central area is asymmetric due to the presence of shorter striae opposite to the stigma. Stigma opening is circular in valve exterior, internally it shows a variety of transversely elongated short, long and sperm shaped openings (Figs. 5.1 and 5.3). The outer fissure of the raphe undulates slightly, but the inner fissure extends straight. The external central endings of the raphe branch are slightly swollen and terminate straight, slightly expanded as well as pore-like deflected or spatulate. The internal central endings are hooked, deflected or have a strongly hooked “parrot head” toward the side of the stigma (Figs. 5.1, 5.3). In both poles, polar fissures are abruptly curved toward the side of the stigma and then extended to the opposite side to terminate. In one side of the valve, the central striae extend long towards the stigma, while on the other side the central striae is very short or even two short striae are observed.

Comment: In order to have micro-morphological as well as molecular data available for this taxon, we have decided to describe this taxon as new and not elevate it from the level of forma to species rank.

***Gomphonema lagenula*** **Kützing** (Figs. 2.26–28; 3.4; 5.6)

Kützing, Die kieselschaligen Bacillarien oder Diatomeen. p.85, pl. 30, fig.60. [25] 1844.

Homotypic synonym: *Gomphonema parvulum* var. *lagenula* (Kützing) Frenguelly 1923.

Lectotype designated by Krammer & Lange-Bertalot [40] 1986: 358ff; fig. 154:8.

Type locality: Trinidad, Tobago.

Epitype (designated here): B 40 0040918 (strain D33_024), see Fig. 2.28

Epitype locality (designated here): Mexico, Ixtlán de los Hervores, 06.12.2004, leg. N. Abarca.

Emended species description:

This taxon is separable from *G. parvulum* based on the shape of the head pole, which is consistently more rostrate to capitate. This taxon is differentiated from *G. parvulum* by its rostrate to capitate valve shape with strongly protruded valve ends and areolae divided by struts in the alveolus. This taxon has a broadly lanceolate shape with markedly rostrate to slightly subcapitate polar ends (Figs. 2.28 and 3.4). Striae vary from parallel in the central area to parallel or slightly convergent at the poles. Within the central area, there is a short stria located opposite to the stigma. This also results in an asymmetrical central area.

***Gomphonema narodoense*** **R. Jahn, Abarca, J. Zimmermann & Enke nov. sp.** (Figs. 2.44–49; 3.9; 4.6; 4.9; 5.2)

Holotype (designated here): B 40 0040917 (strain D23_012); see Fig. 2.45. Type locality: South Korea, DukheungRi, NaeNarodo, spring at rice paddy, 14.10.2004, leg. R. Jahn.

Diagnosis: This taxon is separable from *G. parvulum* based on the shape. Frustule in girdle view narrow rectangular, hardly perceptible cuneate. Lanceolate to linear-lanceolate, almost naviculoid with only moderate rostrate head pole in comparison to the foot-pole (Figs. 2.44–2.47). Length 17–24 µm, breadth 5–6 µm, striae 16–18 in 10 µm, with transverse orientation. Areolae open and extremely variable from small rounded pores to C-, E-, 3- and anchorlike-shapes (Figs. 4.6 and 4.9). The alveoli are oval, elongated and separated by opened silica struts (Fig. 5.2). Stigma has a round opening (Fig. 5.2). Axial area is linear and the central area is narrow. The central area is asymmetric due to the presence of shorter striae opposite the stigma which open circular in valve exterior, internally it shows round openings. The external central endings of the raphe branch terminate straight, slightly expanded as well as pore-like deflected. The internal central endings are hooked toward the side of the stigma. In both poles, polar fissures are abruptly curved toward to the side of the stigma and then extended and terminate towards the opposite side.

### Mantel tests

The Mantel test revealed a positive correlation between genetic and geographic distances separating the *Gomphonema* strains (rm = 0.88; p = 0.001). Also for the central Continental European *Gomphonema* strains there was a positive correlation (rm = 0.84; p = 0.001). No association between geographic and genetic distance was found for the strains within the Faroe Islands (rm = 0.40; p = 0.101).

## Discussion

### Phylogeny and taxonomy

*G. parvulum s.l.* has long been recognized as a highly variable species complex with broad ecological tolerances and many varieties. Many references suggest there is great richness of morphologies that seem to inter-grade with each other and form a morphologically diverse but single taxon [32], [31], [36], [39], [40], [41], [50]. The separation of these morphologies using the valve structure is very difficult. Even though some separation has been done based on slight shape differences, there is great overlap in characters such as dimensions and striae density. Wallace and Patrick [36] considered that the morphological variability in *G. parvulum* is due to the varying conditions of the environment in which these organisms are found, and synonymized many varieties under the single taxon *G. parvulum*. In recent years, studies on the taxonomy of this group have resulted in the splitting off of many new varieties and forms, and old names synonymized in the past have been restored in order to accommodate some of the morphological variations [41]. In addition, many of these varieties have been recognized as sufficiently morphologically different from the nominate variety to be raised to species status [40], [41], [74]. However, other varieties have yet to be examined concerning their micromorphology with SEM or morphometrically assessed to see how morphologically different they are from nominate and other conspecific varieties [75].

The current taxonomic separation of strains within *G. parvulum* s.l. is based on morphological criteria alone. But this current taxonomic concept is only partly supported by our study on the morphological variability of strains (Table 3). Some strains exhibited overlapping morphological features (shape, striation, and stigma). Morphological features such as “attached by a stalk” or “free living” have already been recognized as unreliable [26], [27], [28], [29]. The features shape and striation density of the closely related *Gomphonema augur* Ehrenberg have been recognized to be influenced by environmental conditions [76]. Our results and those of Kermarrec et al. [50] also show that the molecular separation of the taxa does not completely coincide with the morphological taxa, we have therefore called them here morphodemes.

Our combined morphological and molecular results separated at least four taxa/clades clustering geographically (Fig. 7) according to our investigated sites in Korea, Mexico, Central Europe and Northern Atlantic Europe. This is partly supported by Kermarrec et al. [50] who discuss the existence of four or five semi-cryptic taxa in their paper which includes only sites from Europe or the tropical island of Mayotte.

### *Gomphonema parvulum* sensu stricto (Clade 1)

In our study, we found Clade 1 (Fig. 7) to be morphologically and molecularly the most heterogenous clade for which we also had the most strains available. But a main *G. parvulum*–group can be defined because of no or little difference (below one percent) between 11 strains (see details in results). We have chosen D16_045 as the epitype of *Gomphonema parvulum* var. *parvulum*. In this main group though, we found one strain with apparently typical specimens of *G. exilissimum* (D12_022) with characters hardly separable using the light microscope (Fig. 2.24–2.25). But in SEM studies of the cultured material, the main differentiating feature, that the foramina of the areolae are supposed to be significantly larger [61], could not be confirmed. In our molecular results the morphodeme *G. exilissium* was resolved within the main group of *G. parvulum* (Fig. 7); interestingly however, this strain is the only one from the Island Vagar (D12). Kermarrec et al. [50] also show a strain (TCC439) with *exilissimum* features which molecular analyses resolve within their clade A which is restricted to their tropical Island Mayotte. This points at infraspecific variability of morphological features; we believe that *G. exilissimum* should be considered a morphodeme or perhaps an ecodeme if data shows that it only appears in specific oligotrophic waters. *G. exilissimum* is reported as a characteristic species in slightly acid waters with low electrolyte content and low nutrients on silica bedrock for Central Europe [23], [49]. By contrast, Hofmann et al. [49] report the occurrence of *G. parvulum* (var. *parvulum*) in oligosaprobic and mesosaprobic waters independent of their trophic situation. The wide variability of *G. parvulum* Kützing has also been recorded as producing a morphological oddity, the Janus cell [77]. This occurs when two morphologically distinct valves are produced on either side in a single diatom frustule. This phenomenon is considered rare but inducible, and indicates that various phenotypes can be produced by the same genotype. Mann [78] suggests that because the two valves of a diatom frustule form at different times, each may be influenced by different environmental conditions and hence exhibit two different morphological forms.

The three cultures (D16_011, D16_026, D16_028) which do not show up in the main *G. parvulum*-group (Fig. 7) show much morphological and molecular diversity; since some data are missing, they are not completely comparable to the other 11 cultures (Fig. 7). Nevertheless, it is interesting to note how much molecular diversity is present in such a small place since 12 strains (D16) were established from the same sample from Viöoy of the Faröer Islands; the other two are from adjacent islands Sandoy (D13) and Vagar (D12). If this is normal molecular diversity of a taxon, or if this could hint at concealed diversity representing different varieties, or an early stage of an island radiation process, needs to be left for later studies. In the analysis including the additional Genbank sequences (Fig. 8), none of the additional strains but only the 14 strains from the Faroe Islands belong to Clade 1 which in our study represents *Gomphonema parvulum* sensu stricto.

Reported occurrences for *Gomphonema parvulum* are hard to unravel since it has been used as a collective name for the entire species complex for two centuries; being characteristic of organically polluted waters, but also found in samples from a wide range of water qualities [79], [39], [60]. Further studies are needed to determine if this taxon *sensu stricto* is really ubiquitous and cosmopolitan or if it is restricted to specific environments or geographies.

### *Gomphonema saprophilum* (Clade 2)

The three cultures which we have described as *G. saprophilum* form a well-supported clade. Although the three cultures were sampled from three different sites, they are morphologically and molecularly almost homogeneous. This clade has a combined distance of 1.0–1.5% (Appendix S1/C4) from the Faroe clade and might be representative of a northern-central, Continental European clade, since it has been found in waters in Berlin (northern Germany) as well as Skepparkroken, Ängelholm (southern Sweden) which are less than 600 km apart; both waters are highly anthropogenically influenced. In the analysis including the additional Genbank sequences (Fig. 8) two strains originating from France and one from Luxembourg clustered next to our Clade 2 and represent clade C in the corresponding paper [50]. Clade C was identified for Europe only but few other localities were examined in [50] and supports our findings of a (Central) European distribution of this entire clade. Morphologically however, these three strains of clade C [50] do not belong to our concept of *G. saprophilum*; they might represent a different semi-cryptic species.

Since this taxon has only recently been differentiated as *G. parvulum* var. *parvulum* f. *saprophilum* [41], little is known about its occurrence. Hofmann et al. [49] who differentiate it from *G. parvulum* var. *parvulum* f. *parvulum*, report this species for Central Europe as occurring in organically loaded, alpha-mesosaprobe to polysaprobe waters. Kobayasi et al. [42] describe a *Gomphonema parvulum* var. *neosaprophilum* Kobayasi ex K.Osada from a river in Tokyo; whether this is the same taxon or one with a similar morphology needs to be checked.

### *Gomphonema lagenula* (Clade 3)

Although Kützing [26] had described this species from the tropical nation Trinidad and Tobago, the conspecificity of *G. parvulum* and *G. lagenula* has been discussed for a long time. Frenguelli [32], for instance, understood that there were differences between *G. parvulum and G. lagenula*, but these differences were considered by him too subtle to distinguish them as different species. Thus, this author regarded *G. lagenula* as a variety of *G. parvulum*. Since then, most authors have accepted Frenguelli's [32] proposal, differentiating both taxa at infraspecific level [60]. Our results show a well-supported clade (Clade 3, Fig. 6, Fig. 7), separating the Mexican cultures from all the others with 1.2–2.0% difference across all three markers (Appendix S1/C4).

In this study, this taxon has been found in an anthropogenically influenced river. Besides this, it is regularly reported mainly from tropical Central and Southern America [80], [81]; but it has also been reported as occurring in central Europe [60], in Japan [42] in Korean waters [82], and in Burundi (Africa) [83]. Two GenBank sequences from Mayotte Island (Indian Ocean) appear close to this clade (Appendix S1/B7, S1/B8); strain TCC429 is shown in Fig. 8 and belongs to clade A in Kermarrec et al. [50]. The genetic distance between our Mexican strains and TCC429 is 0.5% across all three markers (Appendix S1/C5). The strain TCC500 from Mayotte (not shown in Fig. 8) is molecularly identical with TCC429 in ITS and *rbc*L (Appendix S1/B7, S1/B8) and has been identified as *G.* cf. *lagenula* in Genbank. Since in the corresponding paper [50] clade A is restricted to Mayotte strains, this points at a tropical distribution of this taxon; which is also supported by the morphology of the strains in clade A [50], except for TCC 500 which has very small valves and is therefore difficult to identify. Although Kermarrec et al. [50] did identify one strain as *G.* cf. *lagenula*, their morphological concept as well as their restricted geographical data did not allow them to separate this taxon from their broad *Gomphonema parvulum* complex.

The strain TCC736 from Portugal, sitting on the branch of Clade 3 (Fig. 8) with a distance of 1.3% to the Mexican strains across all three markers (Appendix S1/C5) and representing clade D, is probably a different species. This is also supported by the morphology; we would not have identified this taxon as belonging to *Gomphonema parvulum sensu lato*. Zimmermann et al. [53] have shown that in the well studied *Sellaphora pupula* group a distance between taxa of 0.3% to 0.8% for 18SV4 usually indicates that they are different species. In addition, the ITS marker of TCC736 has an unique insertion of 12 bp; a second insertion of 6 bp found in TCC736 has been found in all members of Clade 1 and Clade 2 plus TCC595, TCC612 and TCC664 and the Mexican strains of Clade 3.

### *Gomphonema narodoense* (Clade 4)

Although this taxon is very hard to differentiate morphologically; it is sufficiently distinct to be supported on these characters alone (Fig. 6). Molecularly, it is a well supported clade clearly separated from the Mexican, the central Continental European and the Faroe Clade with 1.2–2.3% difference across all three markers (Appendix S1/C4). This new taxon has been discovered in a spring on one of the many islands in Southern Korea. It has never been reported before; the recently compiled Algal Flora of Korea [82] lists *Gomphonema parvulum* as very common in Korea, it might be that *G. narodoense* is not restricted to this island but its distribution is hidden within the broad species-complex concept of *G. parvulum*.

The three additional Genbank sequences from Mayotte (TCC492) and Portugal (TCC725, TCC734), named clade B and B′ respectively in the corresponding paper [50], form a sister clade to our Clade 4 because the genetic difference across all three markers between our Korean strains and these strains range between 0.2% and 0.3% (Appendix S1/C5). But when the markers are considered individually, there is no variation in 18SV4 between all five strains (Appendix S1/C7) and for *rbc*L only TCC492 from Mayotte shows 0.7% difference to all the other strains in this clade (Appendix S1/C7). But the ITS morphology of the two sub-clades shows a decisive difference as our Korean strains have an insert of 6 bp which the three TCC-strains from the sister sub-clade do not have. This insert of 6 bp is found in all of our own strains except for Clade 3. Morphologically, only strains TCC492, TCC428 and TCC436 from the clade B, Mayotte, [50] show some resemblance to our concept of *Gomphonema narodoense*; all the other strains of clade B are very different.

Interestingly, this clade, the combined data of our findings (our Clade 4) plus the data of Kermarrec et al [51] (their Clade B & B′) (see Fig. 8), is the most cosmopolitan in this study as it clusters strains from such distances as a South Korean Island in the Western Pacific Ocean with strains from Portugal in southern Europe at the Atlantic Ocean and a tropical strain from Mayotte Island in the Indian Ocean. But this might be due to arbitrary statistics because, although the 18SV4 is the same, the rbcL data show differences; the biggest differences can be found in the ITS data where our Korean strains (plus our other strains except for Mexico) have a 6 bp insert which is missing in the other strains of Kermarrec et al [51], Many more strains from many different localities are needed and have to be studied carefully to determine if we are dealing here with mistaken or missing data simulating a specific distribution pattern.

### Biogeography

Our own findings of four clearly separated taxa (see Fig. 7), partly supported by recent data (see Fig. 8) [50] within the *G. parvulum* complex contradict the hypothesis of cosmopolitanism of microorganisms [1], [3]. Our four well defined clades and taxa, *G. parvulum sensu stricto*, *G. saprophilum*, *G. lagenula*, *and G. narodoense*, which are on average only 25 µm long, are not distributed randomly but hint at a biogeographical or phylogeographical pattern (see also Boo et al. [4]). Although we cannot back it up with detailed water chemistry data, we think that they also do not support the hypothesis that only the environments selects [1], [2]. The tropical Mexican *G. lagenula* and the strains of *G. saprophilum* from the northern-central Continental European clade are from anthropogenically influenced waters; if water quality alone would decide on their occurrence, both should be the same species; this we had originally expected since the polluted Mexican River Lerma had been sampled for comparable European taxa to be used in bio-monitoring. The same argument applies to the Korean *G. narodoense* and the Faroese *G. parvulum sensu strictu* which are both from very clean islands' fresh waters. The difference in climate such as water temperature between tropical Mexico and temperate Europe as well as between islands in the North Atlantic and Western Pacific might also have an influence on clade occurrences [84] but our data - supported by the significant correlation of genetic and geographical distances between the four geographic sites as shown by Mantel tests - implies that in this case geographic distances resulting from historical distributions might be one of the reasons which determined which species is living where. This supports recent findings that diatoms have a biogeography [10], [11], [12], [13]; which is best documented by large “flagship taxa” [15]. Our findings go beyond this, because we are showing that even those species which are much smaller and inconspicuous, and which used to belong to the “250 well-known cosmopolitan species” [16], [84] seem to have a biogeography.

The most important research resources for taxonomic and biodiversity studies on bio geography are well documented strains from different parts of the world, but these are hard to come by as single, living cells need to be isolated in order to establish a culture. However these cultures are essential in order to study the strains micro-morphologically and molecularly in detail, because only in this way can DNA sequences be linked unambiguously to a certain morphology. Our data and those recently collected by Kermarrec et al. [51] show, that there is a great deal more diversity in *Gomphonema parvulum* sensu lato to be discovered around the world. But the question is, if there will ever be enough data available to infer the absence of particular genetic lineages from any geographic area. Nevertheless, further studies on more strains of many more taxa from different parts of the earth will show if our findings are an isolated phenomenon or if they are part of a pattern in diatom biogeography [85].

## Supporting Information

Appendix S1 **S1/A** 0/1-Matrix for valve length and breadth as well as number of striae; **S1/B** Trees of Individual Gene Markers (18SV4, ITS, RBCL); **S1/B1** 18SV4: MP strict consensus tree, LB, PB and PP given, black arrows indicate SAMS support, clades named according to Fig. 7; **S1/B2** ITS: MP strict consensus tree, LB, PB and PP given, black arrows indicate SAMS support, clades named according to Fig. 7; **S1/B3** rbcL: MP strict consensus tree, LB, PB and PP given, black arrows indicate SAMS support, clades named according to Fig. 7; **S1/B4** 18SV4 with Genbank sequences: Bootstrapped (10 000 replicates) MP strict consensus tree, PB given, black arrows indicate SAMS support, clades named according to Fig. 7; **S1/B5** 18SV4 with Genbank sequences: Bootstrapped (10 000 replicates) RAxML best scoring ML tree, LB given, black arrow indicate SAMS support,clades named according to Fig. 7; **S1/B6** 18SV4 with Genbank sequences: Majority rule consensus tree from MrBayes, PP given, black arrows indicate SAMS support, clades named according to Fig. 7; **S1/B7** ITS with Genbank: MP strict consensus tree, LB, PB and PP given, black arrows indicate SAMS support, clades named according to Fig. 7; **S1/B8** rbcL with Genbank: MP strict consensus tree, LB, PB and PP given, black arrows indicate SAMS support, clades named according to Fig. 7; **S1/C** Uncorrected P-Distances; **S1/C1** 18SV4; **S1/C2** ITS; **S1/C3** rbcL; **S1/C4** 18SV, ITS and rbcL combined; **S1/C5** 18SV, ITS and rbcL combined, compared to Genbank sequences; **S1/C6** Clade 2: Individual p-Distances for 18SV, ITS and rbcL compared to Genbank sequences; **S1/C7** Clade 3: Individual p-Distances for 18SV, ITS and rbcL compared to Genbank sequences; **S1/C8** Clade 4: Individual p-Distances for 18SV, ITS and rbcL compared to Genbank sequences.
(PDF)
