# Supplementary material for: Does the Cosmopolitan Diatom Gomphonema parvulum (Kützing) Kützing Have a Biogeography?
Source: PLoS One. 2014 Jan 29;9(1):e86885. doi: 10.1371/journal.pone.0086885 (PMC3906111; doi:10.1371/journal.pone.0086885)
Supplement: Appendix S1 — S1/A 0/1-Matrix for valve length and breadth as well as number of striae; S1/B Trees of Individual Gene Markers (18SV4, ITS, RBCL); S1/B1 18SV4: MP strict consensus tree, LB, PB and PP given, black arrows indicate SAMS support, clades named according to Fig. 7; S1/B2 ITS: MP strict consensus tree, LB, PB and PP given, black arrows indicate SAMS support, clades named according to Fig. 7; S1/B3 rbcL: MP strict consensus tree, LB, PB and PP given, black arrows indicate SAMS support, clades named according to Fig. 7; S1/B4 18SV4 with Genbank sequences: Bootstrapped (10 000 replicates) MP strict consensus tree, PB given, black arrows indicate SAMS support, clades named according to Fig. 7; S1/B5 18SV4 with Genbank sequences: Bootstrapped (10 000 replicates) RAxML best scoring ML tree, LB given, black arrow indicate SAMS support,clades named according to Fig. 7; S1/B6 18SV4 with Genbank sequences: Majority rule consensus tree from MrBayes, PP given, black arrows indicate SAMS support, clades named according to Fig. 7; S1/B7 ITS with Genbank: MP strict consensus tree, LB, PB and PP given, black arrows indicate SAMS support, clades named according to Fig. 7; S1/B8 rbcL with Genbank: MP strict consensus tree, LB, PB and PP given, black arrows indicate SAMS support, clades named according to Fig. 7; S1/C Uncorrected P-Distances; S1/C1 18SV4; S1/C2 ITS; S1/C3 rbcL; S1/C4 18SV, ITS and rbcL combined; S1/C5 18SV, ITS and rbcL combined, compared to Genbank sequences; S1/C6 Clade 2: Individual p-Distances for 18SV, ITS and rbcL compared to Genbank sequences; S1/C7 Clade 3: Individual p-Distances for 18SV, ITS and rbcL compared to Genbank sequences; S1/C8 Clade 4: Individual p-Distances for 18SV, ITS and rbcL compared to Genbank sequences. (PDF) [file pone.0086885.s001.pdf]

APPENDIX

A 0/1-MATRIX FOR VALVE LENGTH AND BREADTH AS WELL AS NUMBER OF STRIAE

Valve length in µm

Strain	17	17,5	18	18,5	19	19,5	20	20,5	21	21,5	22	22,5	23	23,5	24	24,5	25	25,5	26	26,5	27	27,5	28	28,5	29	29,5	30
D12_022	0	0	0	0	0	0	0	0	0	0	0	0	0	0	1	1	1	1	1	1	0	0	0	0	0	0	0
D33_024	0	0	0	0	0	0	0	0	0	0	0	0	0	0	0	0	0	0	1	1	1	0	0	0	0	0	0
D33_006	0	0	0	0	0	0	1	1	1	0	0	0	0	0	0	0	0	0	0	0	0	0	0	0	0	0	0
D23_009	1	1	1	1	0	0	0	0	0	0	0	0	0	0	0	0	0	0	0	0	0	0	0	0	0	0	0
D23_012	0	0	0	0	0	0	0	0	0	0	1	1	1	1	1	0	0	0	0	0	0	0	0	0	0	0	0
D16_042	0	0	0	0	0	0	0	0	0	0	0	0	0	0	0	0	1	1	1	1	1	0	0	0	0	0	0
D16_008	0	0	0	0	0	0	0	0	0	0	0	0	1	1	1	1	1	0	0	0	0	0	0	0	0	0	0
D16_009	0	0	0	0	0	0	0	0	0	0	0	0	1	1	1	1	1	0	0	0	0	0	0	0	0	0	0
D16_004	0	0	0	0	0	0	0	0	0	0	0	0	0	0	1	1	1	1	0	0	0	0	0	0	0	0	0
D16_044	0	0	0	0	0	0	0	0	0	0	0	0	0	0	0	0	0	1	1	1	1	0	0	0	0	0	0
D16_026	0	0	0	0	0	0	0	0	0	0	0	0	0	0	0	0	1	1	1	0	0	0	0	0	0	0	0
D16_027	0	0	0	0	0	0	0	0	0	0	0	0	0	0	1	1	1	0	0	0	0	0	0	0	0	0	0
D16_045	0	0	0	0	0	0	0	0	0	0	1	1	1	1	1	0	0	0	0	0	0	0	0	0	0	0	0
D16_005	0	0	0	0	0	0	0	0	0	0	0	0	0	0	0	1	1	1	1	0	0	0	0	0	0	0	0
D16_011	0	0	0	0	0	0	0	0	0	0	0	0	0	0	0	0	0	0	0	0	0	0	1	1	1	1	1
D16_028	0	0	0	0	0	0	0	0	0	0	0	0	0	0	0	0	0	0	0	0	0	0	1	1	1	0	0
D03_167	0	0	0	0	0	0	0	0	0	0	1	1	1	1	0	0	0	0	0	0	0	0	0	0	0	0	0
D20_026	0	0	0	0	0	0	0	0	0	0	0	0	0	0	0	0	0	1	1	1	1	0	0	0	0	0	0
D36_003	0	0	0	0	0	0	0	0	0	0	0	0	0	0	1	1	1	1	0	0	0	0	0	0	0	0	0
D03_031	0	0	0	0	0	0	0	0	1	1	1	0	0	0	0	0	0	0	0	0	0	0	0	0	0	0	0
D16_030	0	0	0	0	0	0	0	0	0	0	0	0	0	0	0	0	1	1	1	0	0	0	0	0	0	0	0
D13_034	0	0	0	0	0	0	0	0	0	0	0	0	0	0	0	0	0	0	0	0	0	1	1	1	0	0	0

Valve breadth in µm

Strain	5	5,5	6	6,5	7	7,5
D12_022	1	1	1	0	0	0
D33_024	0	0	1	1	1	0
D33_006	0	0	1	1	1	0
D23_009	1	1	1	0	0	0
D23_012	1	1	1	0	0	0
D16_042	0	0	1	1	1	0
D16_008	0	0	1	1	1	0
D16_009	0	0	1	1	1	0
D16_004	0	0	1	1	1	0
D16_044	0	1	1	1	1	0
D16_026	1	1	1	1	1	0
D16_027	0	0	0	0	1	0
D16_045	1	1	1	1	1	0
D16_005	0	0	1	1	1	0
D16_011	0	0	1	1	1	0
D16_028	0	0	1	1	1	0
D03_167	0	1	1	1	0	0
D20_026	0	0	1	1	1	1
D36_003	0	0	1	1	1	1
D03_031	0	0	1	0	0	0
D16_030	1	1	1	0	0	0
D13_034	1	1	1	0	0	0

No Striae

Strain	12	12,5	13	13,5	14	14,5	15	15,5	16	16,5	17	17,5	18	18,5	19	19,5	20	20,5	21
D12_022	0	0	1	1	1	1	1	0	0	0	0	0	0	0	0	0	0	0	0
D33_024	0	0	1	1	1	1	1	1	1	0	0	0	0	0	0	0	0	0	0
D33_006	0	0	0	0	1	1	1	1	1	1	1	1	1	0	0	0	0	0	0
D23_009	0	0	0	0	0	0	0	0	1	1	1	0	0	0	0	0	0	0	0
D23_012	0	0	0	0	0	0	0	0	1	1	1	1	1	0	0	0	0	0	0
D16_042	0	0	0	0	1	1	1	1	1	1	1	1	1	0	0	0	0	0	0
D16_008	0	0	0	0	1	1	1	1	1	1	1	1	1	0	0	0	0	0	0
D16_009	0	0	0	0	1	1	1	1	1	1	1	0	0	0	0	0	0	0	0
D16_004	0	0	0	0	0	0	1	1	1	1	1	1	1	0	0	0	0	0	0
D16_044	0	0	0	0	1	1	1	1	1	1	1	1	1	1	1	1	0	0	0
D16_026	0	0	0	0	1	1	1	1	1	1	1	0	0	0	0	0	0	0	0
D16_027	0	0	0	0	0	0	1	1	1	1	1	0	0	0	0	0	0	0	0
D16_045	1	1	1	1	1	1	1	1	1	1	1	1	1	0	0	0	0	0	0
D16_005	0	0	0	0	1	1	1	0	0	0	0	0	0	0	0	0	0	0	0
D16_011	0	1	1	1	1	1	1	1	1	1	1	1	1	1	1	0	0	0	0
D16_028	0	0	0	0	1	1	1	0	0	0	0	0	0	0	0	0	0	0	0
D03_167	0	0	0	0	1	1	1	1	1	1	1	1	1	1	1	1	1	1	1
D20_026	0	0	1	1	1	1	1	1	1	1	1	0	0	0	0	0	0	0	0
D36_003	0	0	0	0	1	1	1	1	1	1	1	1	1	1	1	1	1	0	0
D03_031	0	0	0	0	0	0	0	0	1	0	0	0	0	0	0	0	0	0	0
D16_030	0	0	0	0	0	0	1	1	1	0	0	0	0	0	0	0	0	0	0
D13_034	0	0	0	0	0	0	1	1	1	0	0	0	0	0	0	0	0	0	0

B TREES OF INDIVIDUAL GENE MARKERS (18SV4, ITS, *RBC*L)

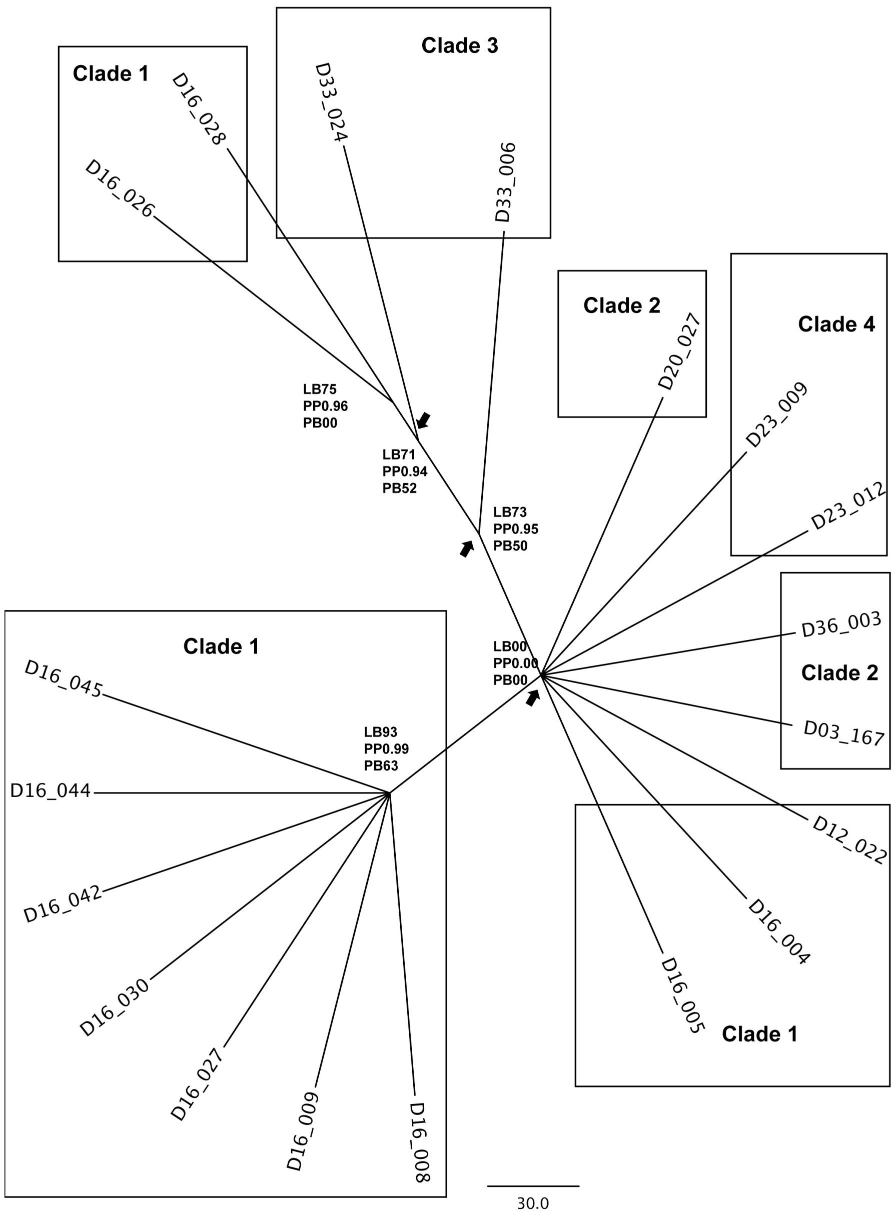

B1 18SV4: MP strict consensus tree, LB, PB and PP given, black arrows indicate SAMS support, clades named according to Fig. 7.

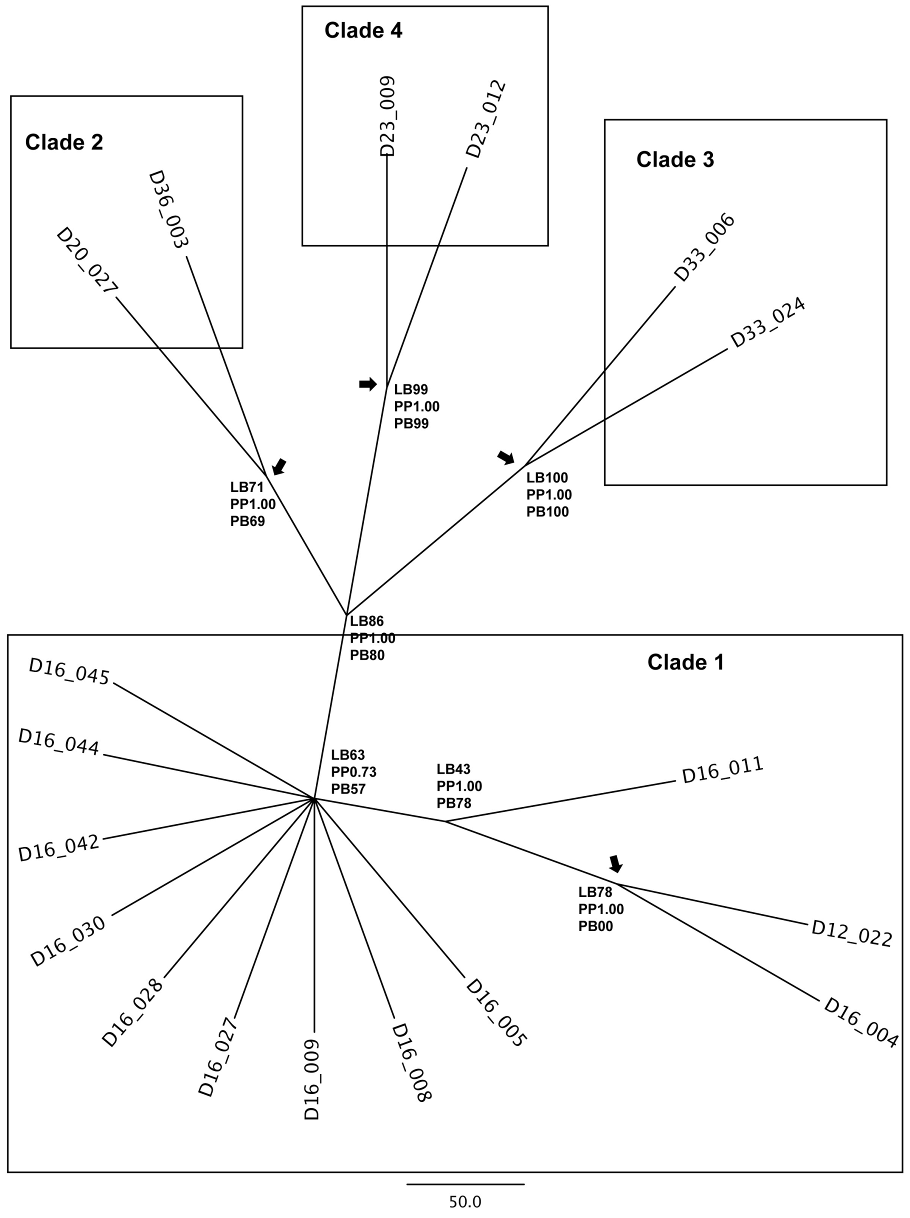

B2 ITS: MP strict consensus tree, LB, PB and PP given, black arrows indicate SAMS support, clades named according to Fig. 7.

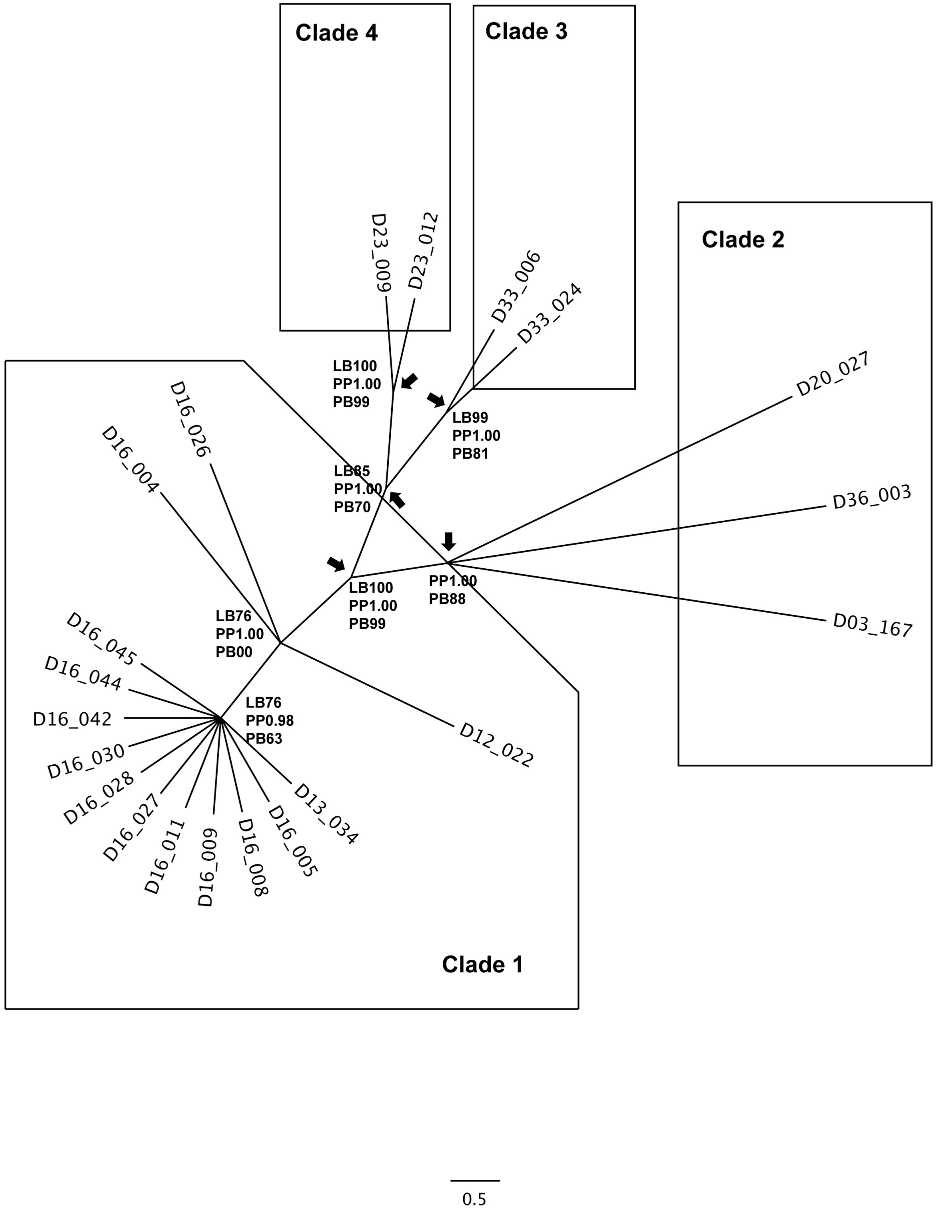

B3 rbcL: MP strict consensus tree, LB, PB and PP given, black arrows indicate SAMS support, clades named according to Fig. 7

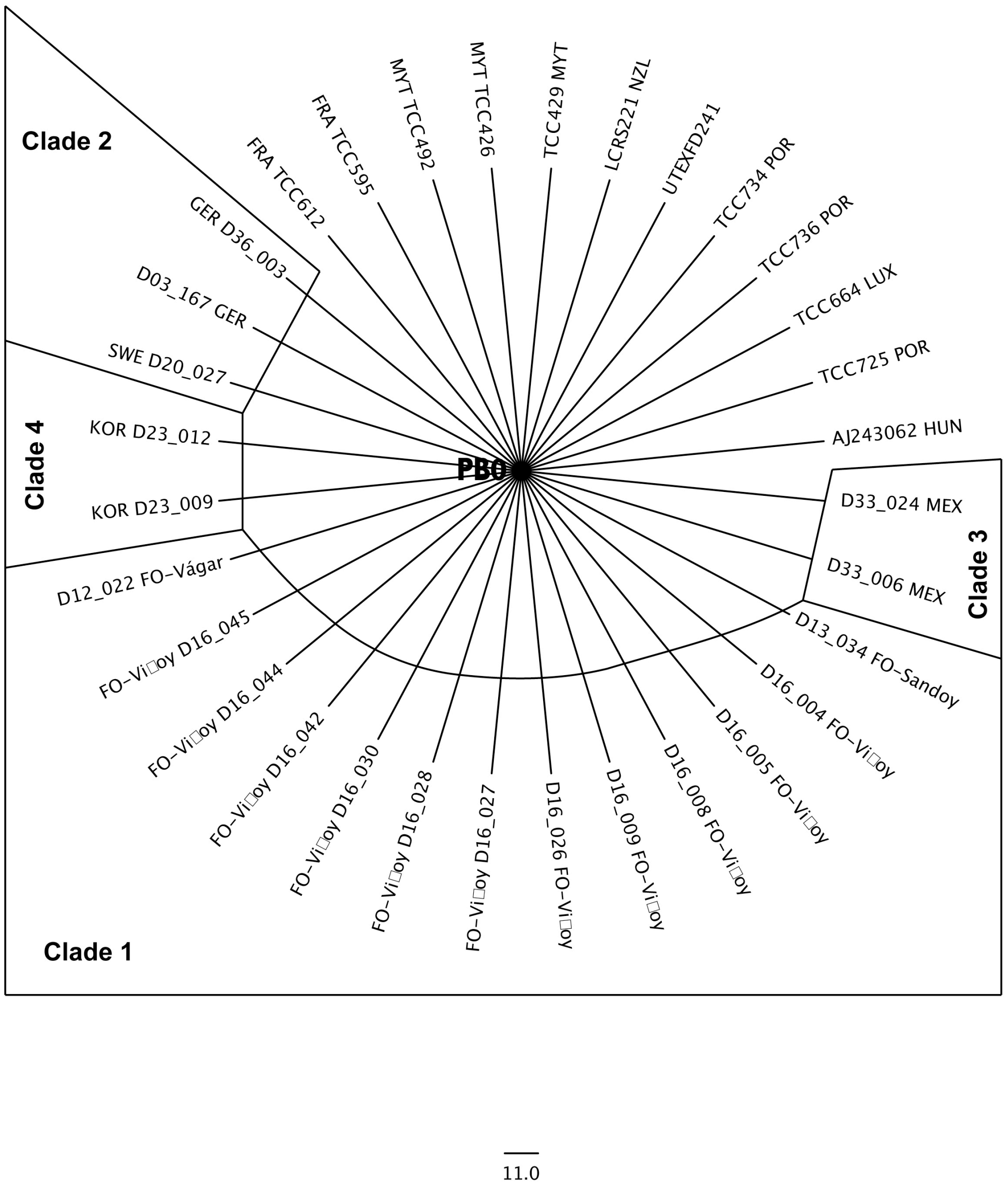

B4 18SV4 with Genbank sequences: Bootstrapped (10 000 replicates) MP strict consensus tree, PB given, black arrows indicate SAMS support, clades named according to Fig. 7

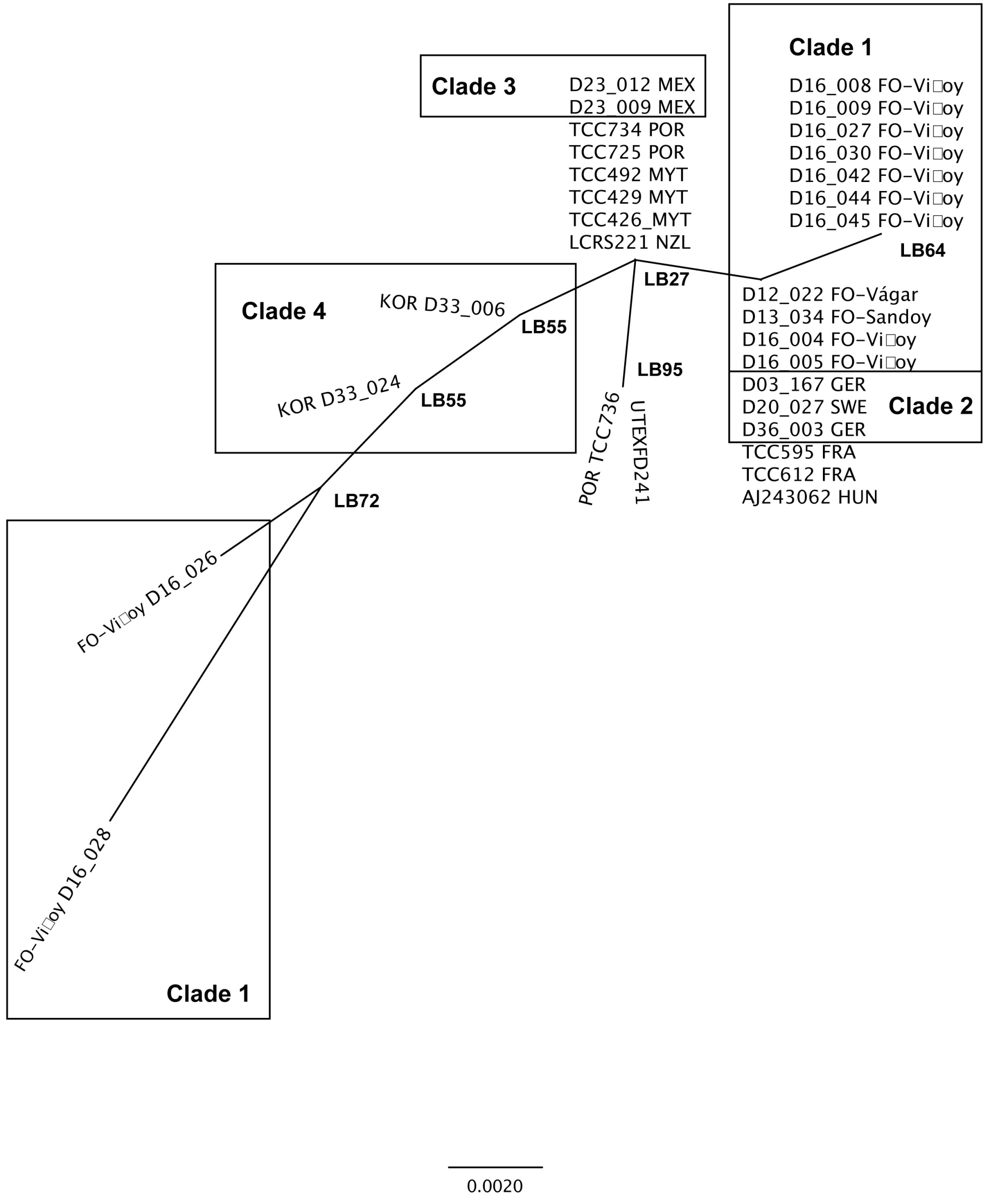

B5 18SV4 with Genbank sequences: Bootstrapped (10 000 replicates) RAxML best scoring ML tree, LB given, black arrow indicate SAMS support,clades named according to Fig. 7

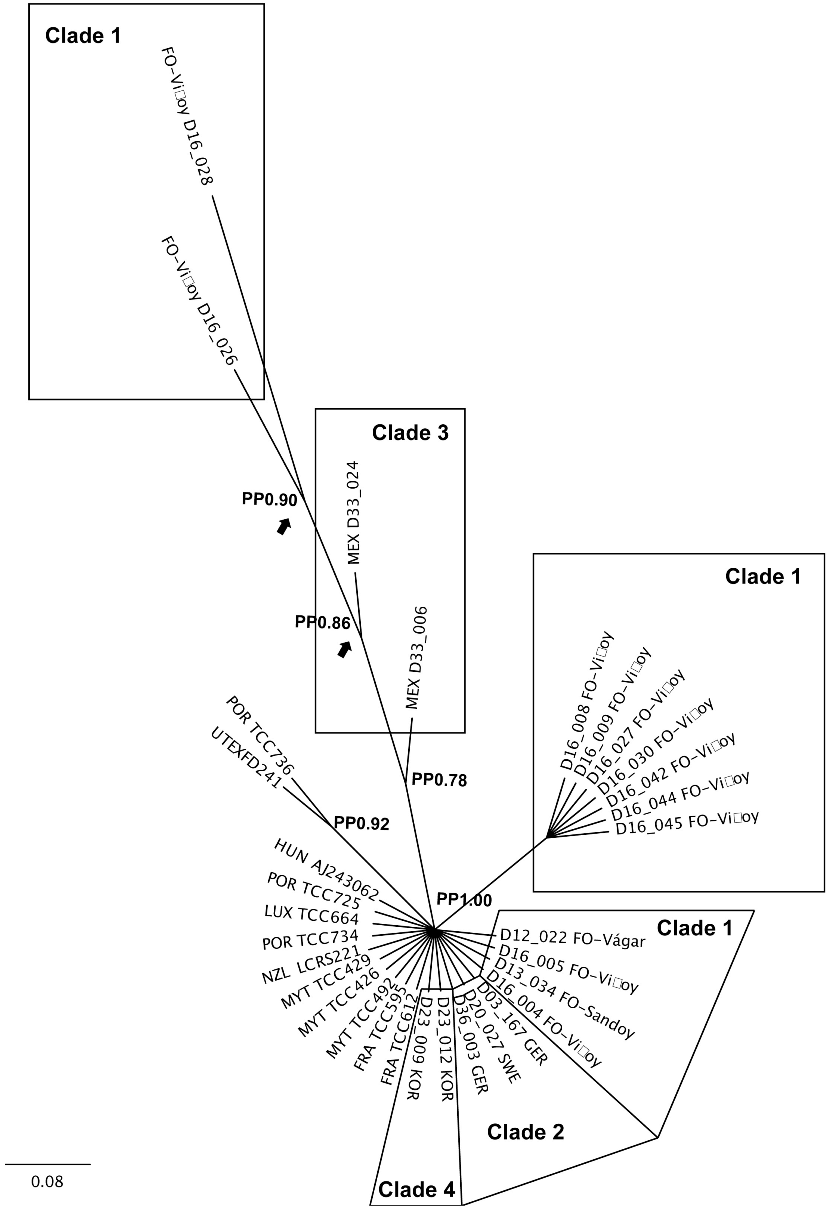

B6 18SV4 with Genbank sequences: Majority rule consensus tree from MrBayes, PP given, black arrows indicate SAMS support, clades named according to Fig. 7

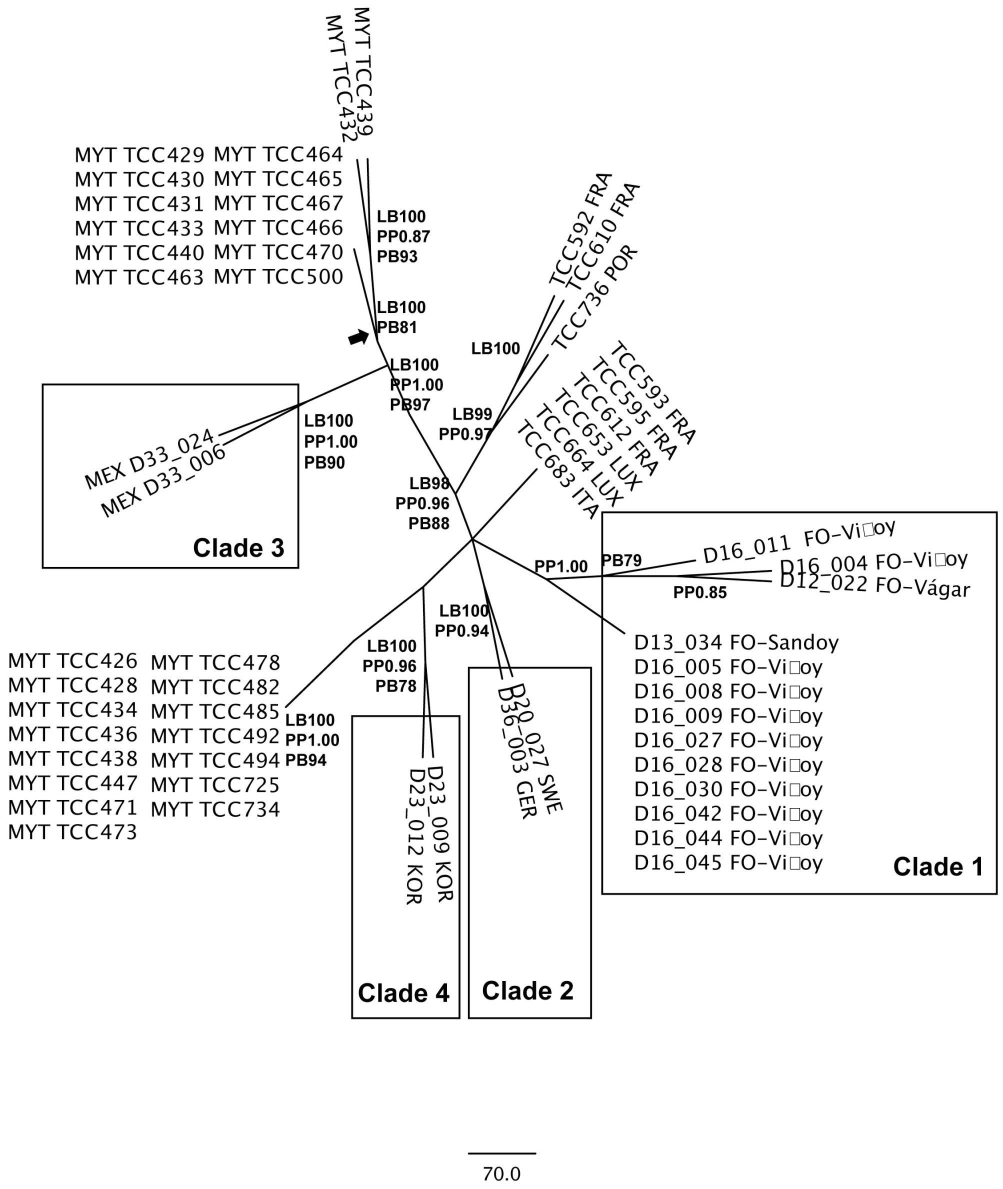

B7 ITS with Genbank: MP strict consensus tree, LB, PB and PP given, black arrows indicate SAMS support, clades named according to Fig. 7.

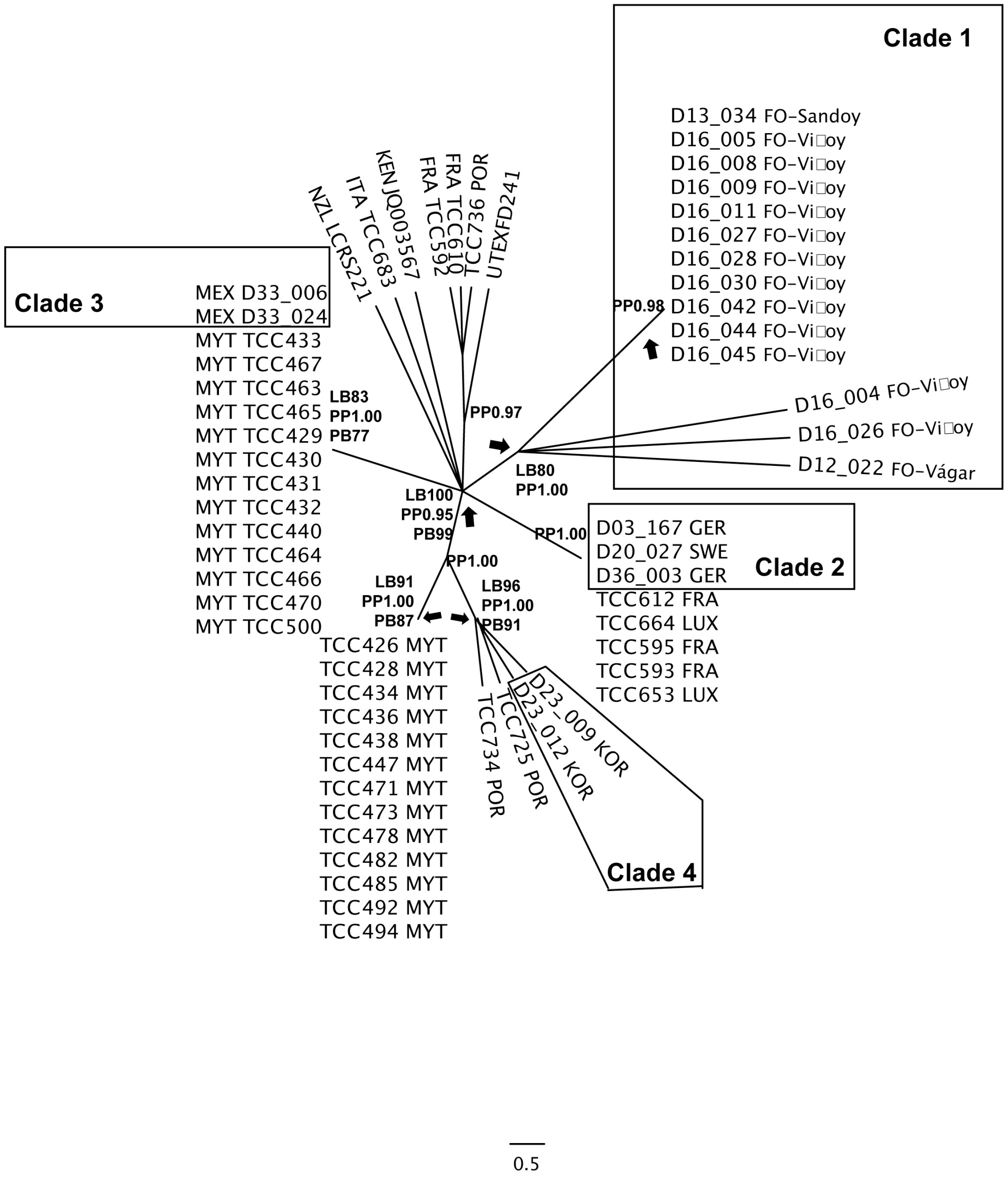

B8 *rbc*L with Genbank: MP strict consensus tree, LB, PB and PP given, black arrows indicate SAMS support, clades named according to Fig. 7.

C UNCORRECTED P-DISTANCES

C1 18SV4

Strain 1	Strain 2	uncorrected p-distance	Strain 1	Strain 2	uncorrected p-distance	Strain 1	Strain 2	uncorrected p-distance	Strain 1	Strain 2	uncorrected p-distance	Strain 1	Strain 2	uncorrected p-distance	Strain 1	Strain 2	uncorrected p-distance	Strain 1	Strain 2	uncorrected p-distance	Strain 1	Strain 2	uncorrected p-distance
D12 022	D03 167	0.0000	D16 027	D16 004	0.0027	D16 042	D12 022	0.0027	D16 045	D16 005	0.0027	D23 009	D12 022	0.0027	D23 012	D16 042	0.0053	D33 024	D16 004	0.0080	D36 003	D16 028	0.0133
D16 004	D03 167	0.0000	D16 027	D16 005	0.0027	D16 042	D16 004	0.0027	D16 045	D16 008	0.0000	D23 009	D16 004	0.0027	D23 012	D16 044	0.0053	D33 024	D16 005	0.0080	D36 003	D16 030	0.0027
D16 004	D12 022	0.0000	D16 027	D16 008	0.0000	D16 042	D16 005	0.0027	D16 045	D16 009	0.0000	D23 009	D16 005	0.0027	D23 012	D16 045	0.0053	D33 024	D16 008	0.0106	D36 003	D16 042	0.0027
D16 005	D03 167	0.0000	D16 027	D16 009	0.0000	D16 042	D16 008	0.0000	D16 045	D16 026	0.0106	D23 009	D16 008	0.0053	D23 012	D20 027	0.0027	D33 024	D16 009	0.0106	D36 003	D16 044	0.0027
D16 005	D12 022	0.0000	D16 027	D16 026	0.0106	D16 042	D16 009	0.0000	D16 045	D16 027	0.0000	D23 009	D16 009	0.0053	D23 012	D23 009	0.0000	D33 024	D16 026	0.0053	D36 003	D16 045	0.0027
D16 005	D16 004	0.0000	D16 028	D03 167	0.0133	D16 042	D16 026	0.0106	D16 045	D16 028	0.0159	D23 009	D16 026	0.0106	D33 006	D03 167	0.0053	D33 024	D16 027	0.0106	D36 003	D20 027	0.0000
D16 008	D03 167	0.0027	D16 028	D12 022	0.0133	D16 042	D16 027	0.0000	D16 045	D16 030	0.0000	D23 009	D16 027	0.0053	D33 006	D12 022	0.0053	D33 024	D16 028	0.0106	D36 003	D23 009	0.0027
D16 008	D12 022	0.0027	D16 028	D16 004	0.0133	D16 042	D16 028	0.0159	D16 045	D16 042	0.0000	D23 009	D16 028	0.0159	D33 006	D16 004	0.0053	D33 024	D16 030	0.0106	D36 003	D23 012	0.0027
D16 008	D16 004	0.0027	D16 028	D16 005	0.0133	D16 042	D16 030	0.0000	D16 045	D16 044	0.0000	D23 009	D16 030	0.0053	D33 006	D16 005	0.0053	D33 024	D16 042	0.0106	D36 003	D33 006	0.0053
D16 008	D16 005	0.0027	D16 028	D16 008	0.0159	D16 044	D03 167	0.0027	D20 027	D03 167	0.0000	D23 009	D16 042	0.0053	D33 006	D16 008	0.0080	D33 024	D16 044	0.0106	D36 003	D33 024	0.0080
D16 009	D03 167	0.0027	D16 028	D16 009	0.0159	D16 044	D12 022	0.0027	D20 027	D12 022	0.0000	D23 009	D16 044	0.0053	D33 006	D16 009	0.0080	D33 024	D16 045	0.0106			
D16 009	D12 022	0.0027	D16 028	D16 026	0.0106	D16 044	D16 004	0.0027	D20 027	D16 004	0.0000	D23 009	D16 045	0.0053	D33 006	D16 026	0.0080	D33 024	D20 027	0.0080			
D16 009	D16 004	0.0027	D16 028	D16 027	0.0159	D16 044	D16 005	0.0027	D20 027	D16 005	0.0000	D23 009	D20 027	0.0027	D33 006	D16 027	0.0080	D33 024	D23 009	0.0053			
D16 009	D16 005	0.0027	D16 030	D03 167	0.0027	D16 044	D16 008	0.0000	D20 027	D16 008	0.0027	D23 012	D03 167	0.0027	D33 006	D16 028	0.0133	D33 024	D23 012	0.0053			
D16 009	D16 008	0.0000	D16 030	D12 022	0.0027	D16 044	D16 009	0.0000	D20 027	D16 009	0.0027	D23 012	D12 022	0.0027	D33 006	D16 030	0.0080	D33 024	D33 006	0.0027			
D16 026	D03 167	0.0080	D16 030	D16 004	0.0027	D16 044	D16 026	0.0106	D20 027	D16 026	0.0080	D23 012	D16 004	0.0027	D33 006	D16 042	0.0080	D36 003	D03 167	0.0000			
D16 026	D12 022	0.0080	D16 030	D16 005	0.0027	D16 044	D16 027	0.0000	D20 027	D16 027	0.0027	D23 012	D16 005	0.0027	D33 006	D16 044	0.0080	D36 003	D12 022	0.0000			
D16 026	D16 004	0.0080	D16 030	D16 008	0.0000	D16 044	D16 028	0.0159	D20 027	D16 028	0.0133	D23 012	D16 008	0.0053	D33 006	D16 045	0.0080	D36 003	D16 004	0.0000			
D16 026	D16 005	0.0080	D16 030	D16 009	0.0000	D16 044	D16 030	0.0000	D20 027	D16 030	0.0027	D23 012	D16 009	0.0053	D33 006	D20 027	0.0053	D36 003	D16 005	0.0000			
D16 026	D16 008	0.0106	D16 030	D16 026	0.0106	D16 044	D16 042	0.0000	D20 027	D16 042	0.0027	D23 012	D16 026	0.0106	D33 006	D23 009	0.0027	D36 003	D16 008	0.0027			
D16 026	D16 009	0.0106	D16 030	D16 027	0.0000	D16 045	D03 167	0.0027	D20 027	D16 044	0.0027	D23 012	D16 027	0.0053	D33 006	D23 012	0.0027	D36 003	D16 009	0.0027			
D16 027	D03 167	0.0027	D16 030	D16 028	0.0159	D16 045	D12 022	0.0027	D20 027	D16 045	0.0027	D23 012	D16 028	0.0159	D33 024	D03 167	0.0080	D36 003	D16 026	0.0080			
D16 027	D12 022	0.0027	D16 042	D03 167	0.0027	D16 045	D16 004	0.0027	D23 009	D03 167	0.0027	D23 012	D16 030	0.0053	D33 024	D12 022	0.0080	D36 003	D16 027	0.0027			

C2 ITS

Strain 1	Strain 2	uncorrected p-distance	Strain 1	Strain 2	uncorrected p-distance	Strain 1	Strain 2	uncorrected p-distance	Strain 1	Strain 2	uncorrected p-distance	Strain 1	Strain 2	uncorrected p-distance	Strain 1	Strain 2	uncorrected p-distance
D16 004	D12 022	0.02401	D16 030	D16 004	0.01715	D16 044	D16 042	0.00000	D23 009	D12 022	0.04979	D23 012	D16 045	0.02058	D36 003	D16 011	0.03951
D16 005	D12 022	0.02401	D16 030	D16 005	0.00343	D16 045	D12 022	0.02058	D23 009	D16 004	0.04628	D23 012	D20 027	0.01891	D36 003	D16 027	0.01371
D16 005	D16 004	0.01372	D16 030	D16 008	0.00000	D16 045	D16 004	0.01715	D23 009	D16 005	0.03258	D23 012	D23 009	0.01195	D36 003	D16 030	0.01371
D16 008	D12 022	0.02058	D16 030	D16 009	0.00000	D16 045	D16 005	0.00343	D23 009	D16 008	0.02917	D33 006	D12 022	0.04505	D36 003	D16 042	0.01371
D16 008	D16 004	0.01715	D16 030	D16 011	0.02916	D16 045	D16 008	0.00000	D23 009	D16 009	0.02917	D33 006	D16 004	0.04330	D36 003	D16 044	0.01371
D16 008	D16 005	0.00343	D16 030	D16 027	0.00000	D16 045	D16 009	0.00000	D23 009	D16 011	0.05836	D33 006	D16 005	0.02955	D36 003	D16 045	0.01371
D16 009	D12 022	0.02058	D16 042	D12 022	0.02058	D16 045	D16 011	0.02916	D23 009	D16 027	0.02917	D33 006	D16 008	0.02606	D36 003	D20 027	0.00513
D16 009	D16 004	0.01715	D16 042	D16 004	0.01715	D16 045	D16 027	0.00000	D23 009	D16 030	0.02917	D33 006	D16 009	0.02606	D36 003	D23 009	0.03266
D16 009	D16 005	0.00343	D16 042	D16 005	0.00343	D16 045	D16 030	0.00000	D23 009	D16 042	0.02917	D33 006	D16 011	0.05583	D36 003	D23 012	0.02407
D16 009	D16 008	0.00000	D16 042	D16 008	0.00000	D16 045	D16 042	0.00000	D23 009	D16 044	0.02917	D33 006	D16 027	0.02606	D36 003	D33 006	0.02940
D16 011	D12 022	0.04631	D16 042	D16 009	0.00000	D16 045	D16 044	0.00000	D23 009	D16 045	0.02917	D33 006	D16 030	0.02606			
D16 011	D16 004	0.04288	D16 042	D16 011	0.02916	D20 027	D12 022	0.02913	D23 009	D20 027	0.02749	D33 006	D16 042	0.02606			
D16 011	D16 005	0.03259	D16 042	D16 027	0.00000	D20 027	D16 004	0.02568	D23 012	D12 022	0.04121	D33 006	D16 044	0.02606			
D16 011	D16 008	0.02916	D16 042	D16 030	0.00000	D20 027	D16 005	0.01198	D23 012	D16 004	0.03769	D33 006	D16 045	0.02606			
D16 011	D16 009	0.02916	D16 044	D12 022	0.02058	D20 027	D16 008	0.00856	D23 012	D16 005	0.02399	D33 006	D20 027	0.02428			
D16 027	D12 022	0.02058	D16 044	D16 004	0.01715	D20 027	D16 009	0.00856	D23 012	D16 008	0.02058	D33 006	D23 009	0.04518			
D16 027	D16 004	0.01715	D16 044	D16 005	0.00343	D20 027	D16 011	0.03780	D23 012	D16 009	0.02058	D33 006	D23 012	0.03303			
D16 027	D16 005	0.00343	D16 044	D16 008	0.00000	D20 027	D16 027	0.00856	D23 012	D16 011	0.04977	D36 003	D12 022	0.03427			
D16 027	D16 008	0.00000	D16 044	D16 009	0.00000	D20 027	D16 030	0.00856	D23 012	D16 027	0.02058	D36 003	D16 004	0.03082			
D16 027	D16 009	0.00000	D16 044	D16 011	0.02916	D20 027	D16 042	0.00856	D23 012	D16 030	0.02058	D36 003	D16 005	0.01713			
D16 027	D16 011	0.02916	D16 044	D16 027	0.00000	D20 027	D16 044	0.00856	D23 012	D16 042	0.02058	D36 003	D16 008	0.01371			
D16 030	D12 022	0.02058	D16 044	D16 030	0.00000	D20 027	D16 045	0.00856	D23 012	D16 044	0.02058	D36 003	D16 009	0.01371			

C3 *rbc*L

Strain 1	Strain 2	uncorrected p-distance	Strain 1	Strain 2	uncorrected p-distance	Strain 1	Strain 2	uncorrected p-distance	Strain 1	Strain 2	uncorrected p-distance	Strain 1	Strain 2	uncorrected p-distance	Strain 1	Strain 2	uncorrected p-distance	Strain 1	Strain 2	uncorrected p-distance	Strain 1	Strain 2	uncorrected p-distance
D12 022	D03 167	0.0102	D16 026	D16 004	0.0000	D16 030	D16 004	0.0010	D16 044	D16 009	0.0000	D20 027	D16 008	0.0122	D23 012	D03 167	0.0102	D33 006	D16 027	0.0132	D33 024	D23 009	0.0071
D13 034	D03 167	0.0112	D16 026	D16 005	0.0010	D16 030	D16 005	0.0000	D16 044	D16 026	0.0010	D20 027	D16 009	0.0122	D23 012	D12 022	0.0142	D33 006	D16 028	0.0132	D33 024	D23 012	0.0071
D13 034	D12 022	0.0010	D16 026	D16 008	0.0010	D16 030	D16 008	0.0000	D16 044	D16 027	0.0000	D20 027	D16 026	0.0102	D23 012	D13 034	0.0152	D33 006	D16 030	0.0132	D33 024	D33 006	0.0000
D16 004	D03 167	0.0102	D16 026	D16 009	0.0010	D16 030	D16 009	0.0000	D16 044	D16 028	0.0000	D20 027	D16 027	0.0122	D23 012	D16 004	0.0142	D33 006	D16 042	0.0132	D36 003	D03 167	0.0000
D16 004	D12 022	0.0000	D16 027	D03 167	0.0112	D16 030	D16 026	0.0010	D16 044	D16 030	0.0000	D20 027	D16 028	0.0122	D23 012	D16 005	0.0152	D33 006	D16 044	0.0132	D36 003	D12 022	0.0102
D16 004	D13 034	0.0010	D16 027	D12 022	0.0010	D16 030	D16 027	0.0000	D16 044	D16 042	0.0000	D20 027	D16 030	0.0122	D23 012	D16 008	0.0152	D33 006	D16 045	0.0132	D36 003	D13 034	0.0112
D16 005	D03 167	0.0112	D16 027	D13 034	0.0000	D16 030	D16 028	0.0000	D16 045	D03 167	0.0112	D20 027	D16 042	0.0122	D23 012	D16 009	0.0152	D33 006	D20 027	0.0081	D36 003	D16 004	0.0102
D16 005	D12 022	0.0010	D16 027	D16 004	0.0010	D16 042	D03 167	0.0112	D16 045	D12 022	0.0010	D20 027	D16 044	0.0122	D23 012	D16 026	0.0132	D33 006	D23 009	0.0071	D36 003	D16 005	0.0112
D16 005	D13 034	0.0000	D16 027	D16 005	0.0000	D16 042	D12 022	0.0010	D16 045	D13 034	0.0000	D20 027	D16 045	0.0122	D23 012	D16 027	0.0152	D33 006	D23 012	0.0071	D36 003	D16 008	0.0112
D16 005	D16 004	0.0010	D16 027	D16 008	0.0000	D16 042	D13 034	0.0000	D16 045	D16 004	0.0010	D23 009	D03 167	0.0102	D23 012	D16 028	0.0152	D33 024	D03 167	0.0061	D36 003	D16 009	0.0112
D16 008	D03 167	0.0112	D16 027	D16 009	0.0000	D16 042	D16 004	0.0010	D16 045	D16 005	0.0000	D23 009	D12 022	0.0142	D23 012	D16 030	0.0152	D33 024	D12 022	0.0122	D36 003	D16 026	0.0091
D16 008	D12 022	0.0010	D16 027	D16 026	0.0010	D16 042	D16 005	0.0000	D16 045	D16 008	0.0000	D23 009	D13 034	0.0152	D23 012	D16 042	0.0152	D33 024	D13 034	0.0132	D36 003	D16 027	0.0112
D16 008	D13 034	0.0000	D16 028	D03 167	0.0112	D16 042	D16 008	0.0000	D16 045	D16 009	0.0000	D23 009	D16 004	0.0142	D23 012	D16 044	0.0152	D33 024	D16 004	0.0122	D36 003	D16 028	0.0112
D16 008	D16 004	0.0010	D16 028	D12 022	0.0010	D16 042	D16 009	0.0000	D16 045	D16 026	0.0010	D23 009	D16 005	0.0152	D23 012	D16 045	0.0152	D33 024	D16 005	0.0132	D36 003	D16 030	0.0112
D16 008	D16 005	0.0000	D16 028	D13 034	0.0000	D16 042	D16 026	0.0010	D16 045	D16 027	0.0000	D23 009	D16 008	0.0152	D23 012	D20 027	0.0112	D33 024	D16 008	0.0132	D36 003	D16 042	0.0112
D16 009	D03 167	0.0112	D16 028	D16 004	0.0010	D16 042	D16 027	0.0000	D16 045	D16 028	0.0000	D23 009	D16 009	0.0152	D23 012	D23 009	0.0000	D33 024	D16 009	0.0132	D36 003	D16 044	0.0112
D16 009	D12 022	0.0010	D16 028	D16 005	0.0000	D16 042	D16 028	0.0000	D16 045	D16 030	0.0000	D23 009	D16 026	0.0132	D33 006	D03 167	0.0061	D33 024	D16 026	0.0112	D36 003	D16 045	0.0112
D16 009	D13 034	0.0000	D16 028	D16 008	0.0000	D16 042	D16 030	0.0000	D16 045	D16 042	0.0000	D23 009	D16 027	0.0152	D33 006	D12 022	0.0122	D33 024	D16 027	0.0132	D36 003	D20 027	0.0020
D16 009	D16 004	0.0010	D16 028	D16 009	0.0000	D16 044	D03 167	0.0112	D16 045	D16 044	0.0000	D23 009	D16 028	0.0152	D33 006	D13 034	0.0132	D33 024	D16 028	0.0132	D36 003	D23 009	0.0102
D16 009	D16 005	0.0000	D16 028	D16 026	0.0010	D16 044	D12 022	0.0010	D20 027	D03 167	0.0020	D23 009	D16 030	0.0152	D33 006	D16 004	0.0122	D33 024	D16 030	0.0132	D36 003	D23 012	0.0102
D16 009	D16 008	0.0000	D16 028	D16 027	0.0000	D16 044	D13 034	0.0000	D20 027	D12 022	0.0112	D23 009	D16 042	0.0152	D33 006	D16 005	0.0132	D33 024	D16 042	0.0132	D36 003	D33 006	0.0061
D16 026	D03 167	0.0091	D16 030	D03 167	0.0112	D16 044	D16 004	0.0010	D20 027	D13 034	0.0122	D23 009	D16 044	0.0152	D33 006	D16 008	0.0132	D33 024	D16 044	0.0132	D36 003	D33 024	0.0061
D16 026	D12 022	0.0000	D16 030	D12 022	0.0010	D16 044	D16 005	0.0000	D20 027	D16 004	0.0112	D23 009	D16 045	0.0152	D33 006	D16 009	0.0132	D33 024	D16 045	0.0132			
D16 026	D13 034	0.0010	D16 030	D13 034	0.0000	D16 044	D16 008	0.0000	D20 027	D16 005	0.0122	D23 009	D20 027	0.0112	D33 006	D16 026	0.0112	D33 024	D20 027	0.0081			

C4 18SV, ITS and *rbc*L combined

Strain 1	Strain 2	uncorrected p-distances	Strain 1	Strain 2	uncorrected p-distances	Strain 1	Strain 2	uncorrected p-distances	Strain 1	Strain 2	uncorrected p-distances	Strain 1	Strain 2	uncorrected p-distances
D16 004	D12 022	0.0072	D16 042	D16 004	0.0062	D16 045	D16 044	0.0000	D23 012	D12 022	0.0200	D33 006	D20 027	0.0124
D16 005	D12 022	0.0077	D16 042	D16 005	0.0015	D20 027	D12 022	0.0144	D23 012	D16 004	0.0190	D33 006	D23 009	0.0175
D16 005	D16 004	0.0046	D16 042	D16 008	0.0000	D20 027	D16 004	0.0134	D23 012	D16 005	0.0154	D33 006	D23 012	0.0139
D16 008	D12 022	0.0072	D16 042	D16 009	0.0000	D20 027	D16 005	0.0098	D23 012	D16 008	0.0149	D36 003	D12 022	0.0154
D16 008	D16 004	0.0062	D16 042	D16 027	0.0000	D20 027	D16 008	0.0092	D23 012	D16 009	0.0149	D36 003	D16 004	0.0144
D16 008	D16 005	0.0015	D16 042	D16 030	0.0000	D20 027	D16 009	0.0092	D23 012	D16 027	0.0149	D36 003	D16 005	0.0108
D16 009	D12 022	0.0072	D16 044	D12 022	0.0072	D20 027	D16 027	0.0092	D23 012	D16 030	0.0149	D36 003	D16 008	0.0103
D16 009	D16 004	0.0062	D16 044	D16 004	0.0062	D20 027	D16 030	0.0092	D23 012	D16 042	0.0149	D36 003	D16 009	0.0103
D16 009	D16 005	0.0015	D16 044	D16 005	0.0015	D20 027	D16 042	0.0092	D23 012	D16 044	0.0149	D36 003	D16 027	0.0103
D16 009	D16 008	0.0000	D16 044	D16 008	0.0000	D20 027	D16 044	0.0092	D23 012	D16 045	0.0149	D36 003	D16 030	0.0103
D16 027	D12 022	0.0072	D16 044	D16 009	0.0000	D20 027	D16 045	0.0092	D23 012	D20 027	0.0118	D36 003	D16 042	0.0103
D16 027	D16 004	0.0062	D16 044	D16 027	0.0000	D23 009	D12 022	0.0226	D23 012	D23 009	0.0036	D36 003	D16 044	0.0103
D16 027	D16 005	0.0015	D16 044	D16 030	0.0000	D23 009	D16 004	0.0216	D33 006	D12 022	0.0206	D36 003	D16 045	0.0103
D16 027	D16 008	0.0000	D16 044	D16 042	0.0000	D23 009	D16 005	0.0180	D33 006	D16 004	0.0201	D36 003	D20 027	0.0026
D16 027	D16 009	0.0000	D16 045	D12 022	0.0072	D23 009	D16 008	0.0175	D33 006	D16 005	0.0165	D36 003	D23 009	0.0154
D16 030	D12 022	0.0072	D16 045	D16 004	0.0062	D23 009	D16 009	0.0175	D33 006	D16 008	0.0160	D36 003	D23 012	0.0129
D16 030	D16 004	0.0062	D16 045	D16 005	0.0015	D23 009	D16 027	0.0175	D33 006	D16 009	0.0160	D36 003	D33 006	0.0129
D16 030	D16 005	0.0015	D16 045	D16 008	0.0000	D23 009	D16 030	0.0175	D33 006	D16 027	0.0160			
D16 030	D16 008	0.0000	D16 045	D16 009	0.0000	D23 009	D16 042	0.0175	D33 006	D16 030	0.0160			
D16 030	D16 009	0.0000	D16 045	D16 027	0.0000	D23 009	D16 044	0.0175	D33 006	D16 042	0.0160			
D16 030	D16 027	0.0000	D16 045	D16 030	0.0000	D23 009	D16 045	0.0175	D33 006	D16 044	0.0160			
D16 042	D12 022	0.0072	D16 045	D16 042	0.0000	D23 009	D20 027	0.0144	D33 006	D16 045	0.0160			

C5 18SV, ITS and *rbc*L combined, compared to Genbank sequences

Strain 1	Strain 2	uncorrected p-distance	Strain 1	Strain 2	uncorrected p-distance	Strain 1	Strain 2	uncorrected p-distance	Strain 1	Strain 2	uncorrected p-distance	Strain 1	Strain 2	uncorrected p-distance
TCC429	D12 022	0.0231	TCC492	D16 030	0.0164	TCC612	D33 006	0.0123	TCC725	D12 022	0.0200	TCC736	D16 005	0.0133
TCC429	D13 034	0.0185	TCC492	D16 042	0.0164	TCC612	D36 003	0.0036	TCC725	D13 034	0.0159	TCC736	D16 008	0.0128
TCC429	D16 004	0.0221	TCC492	D16 044	0.0164	TCC612	TCC429	0.0138	TCC725	D16 004	0.0195	TCC736	D16 009	0.0128
TCC429	D16 005	0.0185	TCC492	D16 045	0.0164	TCC612	TCC492	0.0133	TCC725	D16 005	0.0159	TCC736	D16 027	0.0128
TCC429	D16 008	0.0179	TCC492	D20 027	0.0134	TCC664	D12 022	0.0138	TCC725	D16 008	0.0154	TCC736	D16 030	0.0128
TCC429	D16 009	0.0179	TCC492	D23 012	0.0056	TCC664	D13 034	0.0092	TCC725	D16 009	0.0154	TCC736	D16 042	0.0128
TCC429	D16 027	0.0179	TCC492	D33 006	0.0154	TCC664	D16 004	0.0128	TCC725	D16 027	0.0154	TCC736	D16 044	0.0128
TCC429	D16 030	0.0179	TCC492	D36 003	0.0139	TCC664	D16 005	0.0092	TCC725	D16 030	0.0154	TCC736	D16 045	0.0128
TCC429	D16 042	0.0179	TCC492	TCC429	0.0139	TCC664	D16 008	0.0087	TCC725	D16 042	0.0154	TCC736	D20 027	0.0113
TCC429	D16 044	0.0179	TCC612	D12 022	0.0143	TCC664	D16 009	0.0087	TCC725	D16 044	0.0154	TCC736	D23 012	0.0113
TCC429	D16 045	0.0179	TCC612	D13 034	0.0097	TCC664	D16 027	0.0087	TCC725	D16 045	0.0154	TCC736	D33 006	0.0128
TCC429	D20 027	0.0143	TCC612	D16 004	0.0133	TCC664	D16 030	0.0087	TCC725	D20 027	0.0123	TCC736	D36 003	0.0118
TCC429	D23 012	0.0129	TCC612	D16 005	0.0097	TCC664	D16 042	0.0087	TCC725	D23 012	0.0026	TCC736	TCC429	0.0107
TCC429	D33 006	0.0046	TCC612	D16 008	0.0092	TCC664	D16 044	0.0087	TCC725	D33 006	0.0144	TCC736	TCC492	0.0118
TCC429	D36 003	0.0143	TCC612	D16 009	0.0092	TCC664	D16 045	0.0087	TCC725	D36 003	0.0128	TCC736	TCC612	0.0102
TCC492	D12 022	0.0211	TCC612	D16 027	0.0092	TCC664	D20 027	0.0020	TCC725	TCC429	0.0129	TCC736	TCC664	0.0097
TCC492	D13 034	0.0169	TCC612	D16 030	0.0092	TCC664	D23 012	0.0113	TCC725	TCC492	0.0031	TCC736	TCC725	0.0118
TCC492	D16 004	0.0205	TCC612	D16 042	0.0092	TCC664	D33 006	0.0118	TCC725	TCC612	0.0123			
TCC492	D16 005	0.0169	TCC612	D16 044	0.0092	TCC664	D36 003	0.0031	TCC725	TCC664	0.0118			
TCC492	D16 008	0.0164	TCC612	D16 045	0.0092	TCC664	TCC429	0.0133	TCC736	D12 022	0.0190			
TCC492	D16 009	0.0164	TCC612	D20 027	0.0026	TCC664	TCC492	0.0128	TCC736	D13 034	0.0133			
TCC492	D16 027	0.0164	TCC612	D23 012	0.0118	TCC664	TCC612	0.0005	TCC736	D16 004	0.0169			

C6 Clade 2**:** Individual p-Distances for 18SV, ITS and *rbc*L compared to Genbank sequences

18S

Strain 1	Strain 2	uncorrected p-Distance
D36 003	D20 027	0.0000
D03 167	D20 027	0.0000
D03 167	D36 003	0.0000
TCC595	D20 027	0.0000
TCC595	D36 003	0.0000
TCC595	D03 167	0.0000
TCC612	D20 027	0.0000
TCC612	D36 003	0.0000
TCC612	D03 167	0.0000
TCC612	TCC595	0.0000
TCC664	D20 027	0.0000
TCC664	D36 003	0.0000
TCC664	D03 167	0.0000
TCC664	TCC595	0.0000
TCC664	TCC612	0.0000

ITS

Strain 1	Strain 2	uncorrected p-Distance
D36 003	D20 027	0.0064
TCC664	D20 027	0.0032
TCC664	D36 003	0.0097
TCC612	D20 027	0.0048
TCC612	D36 003	0.0113
TCC612	TCC664	0.0016
TCC595	D20 027	0.0032
TCC595	D36 003	0.0097
TCC595	TCC664	0.0000
TCC595	TCC612	0.0016

rbcL

Strain 1	Strain 2	uncorrected p-Distance
D20 027	D03 167	0.0022
D36 003	D03 167	0.0000
D36 003	D20 027	0.0022
TCC595	D03 167	0.0000
TCC595	D20 027	0.0022
TCC595	D36 003	0.0000
TCC612	D03 167	0.0011
TCC612	D20 027	0.0032
TCC612	D36 003	0.0011
TCC612	TCC595	0.0011
TCC664	D03 167	0.0000
TCC664	D20 027	0.0022
TCC664	D36 003	0.0000
TCC664	TCC595	0.0000
TCC664	TCC612	0.0011

C7 Clade 3**:** Individual p-Distances for 18SV, ITS and *rbc*L compared to Genbank sequences

18S

Strain 1	Strain 2	uncorrected p-distance
D33 024	D33 006	0.0027
TCC429	D33 006	0.0027
TCC429	D33 024	0.0053
TCC736	D33 006	0.0053
TCC736	D33 024	0.0080
TCC736	TCC429	0.0027

ITS

Strain 1	Strain 2	uncorrected p-distance
TCC429	D33 006	0.0143
TCC736	D33 006	0.0333
TCC736	TCC429	0.0263

rbcL

Strain 1	Strain 2	uncorrected p-distance
D33 024	D33 006	0.0000
TCC429	D33 006	0.0000
TCC429	D33 024	0.0000
TCC736	D33 006	0.0061
TCC736	D33 024	0.0061
TCC736	TCC429	0.0061

C8 Clade 4**:** Individual p-Distances for 18SV, ITS and *rbc*L compared to Genbank sequences

18S			ITS			rbcL		
Strain 1	Strain 2	uncorrected p-distance	Strain 1	Strain 2	uncorrected p-distance	Strain 1	Strain 2	uncorrected p-distance
D23 012	D23 009	0.0000	D23 012	D23 009	0.0119	D23 012	D23 009	0.0000
TCC492	D23 009	0.0000	TCC734	D23 009	0.0172	TCC492	D23 009	0.0065
TCC492	D23 012	0.0000	TCC734	D23 012	0.0086	TCC492	D23 012	0.0065
TCC734	D23 009	0.0000	TCC725	D23 009	0.0172	TCC725	D23 009	0.0000
TCC734	D23 012	0.0000	TCC725	D23 012	0.0086	TCC725	D23 012	0.0000
TCC734	TCC492	0.0000	TCC725	TCC734	0.0000	TCC725	TCC492	0.0065
TCC725	D23 009	0.0000	TCC492	D23 009	0.0172	TCC734	D23 009	0.0000
TCC725	D23 012	0.0000	TCC492	D23 012	0.0086	TCC734	D23 012	0.0000
TCC725	TCC492	0.0000	TCC492	TCC734	0.0000	TCC734	TCC492	0.0065
TCC725	TCC734	0.0000	TCC492	TCC725	0.0000	TCC734	TCC725	0.0000
